# Next generation high throughput DNA damage detection platform for genotoxic compound screening

**DOI:** 10.1038/s41598-018-20995-w

**Published:** 2018-02-09

**Authors:** Peter Sykora, Kristine L. Witt, Pooja Revanna, Stephanie L. Smith-Roe, Jonathan Dismukes, Donald G. Lloyd, Bevin P. Engelward, Robert W. Sobol

**Affiliations:** 1 Department of Oncologic Sciences, Mitchell Cancer Institute, University of South Alabama, 1660 Springhill Avenue, Mobile, AL 36604 USA; 2 Division of the National Toxicology Program, National Institute of Environmental Health Sciences, Research Triangle Park, NC 27709, USA; 3 DGL-Imaging, Millersville, MD 21108 USA; 4 Department of Biological Engineering, MIT, Cambridge, MA 02139 USA

## Abstract

Methods for quantifying DNA damage, as well as repair of that damage, in a high-throughput format are lacking. Single cell gel electrophoresis (SCGE; comet assay) is a widely-used method due to its technical simplicity and sensitivity, but the standard comet assay has limitations in reproducibility and throughput. We have advanced the SCGE assay by creating a 96-well hardware platform coupled with dedicated data processing software (CometChip Platform). Based on the original cometchip approach, the CometChip Platform increases capacity ~200 times over the traditional slide-based SCGE protocol, with excellent reproducibility. We tested this platform in several applications, demonstrating a broad range of potential uses including the routine identification of DNA damaging agents, using a 74-compound library provided by the National Toxicology Program. Additionally, we demonstrated how this tool can be used to evaluate human populations by analysis of peripheral blood mononuclear cells to characterize susceptibility to genotoxic exposures, with implications for epidemiological studies. In summary, we demonstrated a high level of reproducibility and quantitative capacity for the CometChip Platform, making it suitable for high-throughput screening to identify and characterize genotoxic agents in large compound libraries, as well as for human epidemiological studies of genetic diversity relating to DNA damage and repair.

## Introduction

There is compelling evidence that genomic instability plays a prominent role in the initiation of carcinogenesis and it has also been linked to aging as well as to a variety of adverse health conditions such as neurodegenerative syndromes and birth defects (for reviews^1,2^). To combat the effect of DNA damage, cells have evolved multiple, often overlapping DNA repair pathways to ensure that damage is efficiently and accurately repaired. Hence, the ability to measure both endogenous levels of DNA damage and genotoxicant-induced DNA damage is particularly important. Diverse methods for measuring genomic damage have been developed including alkaline unwinding^3^, DNA fiber analysis^4^, direct-damage microscopy^5^ and long amplification PCR^6^. However, all the methods developed thus far have shortcomings, including challenges to be scaled up to a high-throughput format, and a laborious work-flow that makes DNA damage quantification challenging and often difficult to accurately reproduce.

Single cell gel electrophoresis (SCGE), also known as the comet assay, has been used to measure DNA damage in cells or whole organisms for over thirty years^7^. Widely embraced in toxicology and molecular biology, the technique can be used to measure DNA damage and repair in mammalian tissues and cell culture models. Some regulatory agencies consider data from the cell culture-based *in vitro* comet assay when submitted as an addendum to other genotoxicity assays. However, to date, only the *in vivo* comet assay has been adopted by regulatory agencies (in Japan and Europe) as an approach for genotoxicity testing^8^. The theory governing the comet assay is that genotoxicants can induce DNA damage in the form of single-strand breaks, AP sites, and alkali labile sites or adducts that convert to DNA strand breaks under alkali treatment. For an undamaged cell, the DNA is highly supercoiled and upon dissolution of the nuclear membrane, DNA does not migrate significantly through a matrix such as agarose. For a damaged cell, fragmented DNA can more readily migrate and single strand breaks can release super-helical tension, allowing for loops of DNA to migrate toward a positively charged anode. The image of the migrated DNA resembles a comet, from which the assay gets its name. The comet assay also has fewer technical challenges as compared to other protocols such as long amplification-PCR^9^, fluorescence *in-situ* hybridization (FISH)^10^ or the Fluorimetric Detection of Alkaline DNA Unwinding (FADU) assay^11^. However, for all the positive attributes of the comet assay, there remain features that limit its widespread application, despite decades of refinement^12^.

A frequent criticism of the comet assay is the lack of reproducibility. This has directly affected the ability of researchers to compare results to those previously published, a problem highlighted by numerous publications citing differences in inter-laboratory as well as intra-laboratory results^13–17^. The European Standards Committee on Oxidative DNA Damage (ESCODD) has conducted two studies and reported a coefficient of variation (CV) of 57%^18^ and 66%^19^ between research groups given the same biological samples in which to measure DNA damage levels using the assay. Each trial encompassed eight^14^, twelve^13,16^ and ten^17^ different laboratories, respectively. In all, 30 different trials were conducted in the three studies using laboratories at different locations. In the most extreme cases, the differences in the amounts of DNA damage that were measured were as high as 6-fold (also reviewed^20^). This level of variation has ramifications when evaluating DNA damage levels in subjects from different geographical regions as a part of large-scale collaborative studies, making it impossible to distinguish real population differences from inter/intra-laboratory variability.

A significant step in addressing some of the tractable problems associated with the standard comet assay was the development of a microwell system that allowed trapping of single cells^21^. The micro-patterned agarose array allowed cells to be loaded into individual wells, achieving a uniform cell distribution in a single focal plane. This advancement over the standard, slide-based comet methodology ensured non-overlapping comets and simplified image collection, two characteristics amenable to automated image acquisition. The first-generation microwell cometchip system^21,22^ was tested against the slide-based comet assay and results showed that this new approach greatly reduced inter-comet variation^23^. The cometchip has proven to be useful in a number of contexts, including nanoparticle toxicity testing, analysis of DNA double-strand breaks, and studies of DNA methylation status^24–26^. Here, based on the prior successes of the cometchip methodology, we have created a novel CometChip Platform, which offers several novel enhancements over the initial microwell cometchip system^21,22^, making it more robust, versatile, and easy to use, and thus suitable for broad adoption for laboratory, pre-clinical, and population sciences.

Foremost, we have significantly improved the engineering of the platform by introducing a rigid glass support for the gel. Further, we created a 96-well plate “macrowell former” to improve handling, and we developed a dedicated electrophoretic system to limit intra-assay variability. We also employ image acquisition using an automated cytometer, which necessitated the creation of a novel data analysis software package able to accelerate data management, and accurately and efficiently process the high volumes of data generated by the CometChip Platform. Importantly, this novel software includes a user-friendly graphical user interface (GUI), and has been designed to recognize and analyze data from any imaging platform. Having an easy-to-use data analysis program that is cross-compatible among imaging platforms is a critical advance that enables broad usage.

Here, we have evaluated the performance of the CometChip Platform with regard to inter- and intra-assay variability, quantitation of DNA damage and repair, and ability to detect small changes in DNA damage. The CometChip proves to be a robust platform with outstanding data reproducibility, comparable to or in some instances improved over the original cometchip^23^. One advantage is that the design enables use of all 96 wells, which is a limitation in the original cometchip due to the use of clips. Reduced experimental variation (compared to the slide-based comet assay) and a larger sample size (compared to the cometchip) enables experiments that require precision and increased throughput. As examples, increased precision makes possible precise measurement of endogenous levels of DNA damage, and higher throughput enables multiple doses as well as multiple time points, which are needed for evaluating DNA repair kinetics.

We also show that this new CometChip Platform is well-suited for screening large sets of compounds to identify DNA damaging agents. To that end, we screened a panel of compounds provided and sourced by the U.S. National Toxicology Program (NTP). Using the CometChip, we correctly identified a selection of DNA damaging agents from the NTP compound panel in a proof-of-principle experiment. Further, we demonstrated how this new CometChip Platform can be used to evaluate human populations by analysis of peripheral blood mononuclear cells to characterize susceptibility to genotoxic exposures, with implications for epidemiological studies.

## Results

### CometChip apparatus

The CometChip Platform utilizes the basic principles of single cell gel electrophoresis (SCGE). DNA, which has a negative charge, will migrate in an agarose matrix towards the positive anode. In the nucleus of a cell, the DNA is supercoiled and bound to the nuclear matrix, restricting the ability to migrate (Fig. S1A, left). With damage, fragmented DNA can migrate more easily and the supercoiled DNA transitions to a more relaxed state, enhancing migration (Fig. S1A, right). The original cometchip^21^ employed a thin layer of agarose supported on flexible GelBond™ (Lonza, Allendale, NJ) which was not compatible with some existing imaging cytometers. Therefore, we introduced a glass support to replace GelBond™ and created a chemical treatment process to secure the agarose layer to the surface of the glass throughout the assay procedure, thus avoiding agarose gel detachment, as occurs sometimes in the slide-based comet assay.

**Figure 1:**
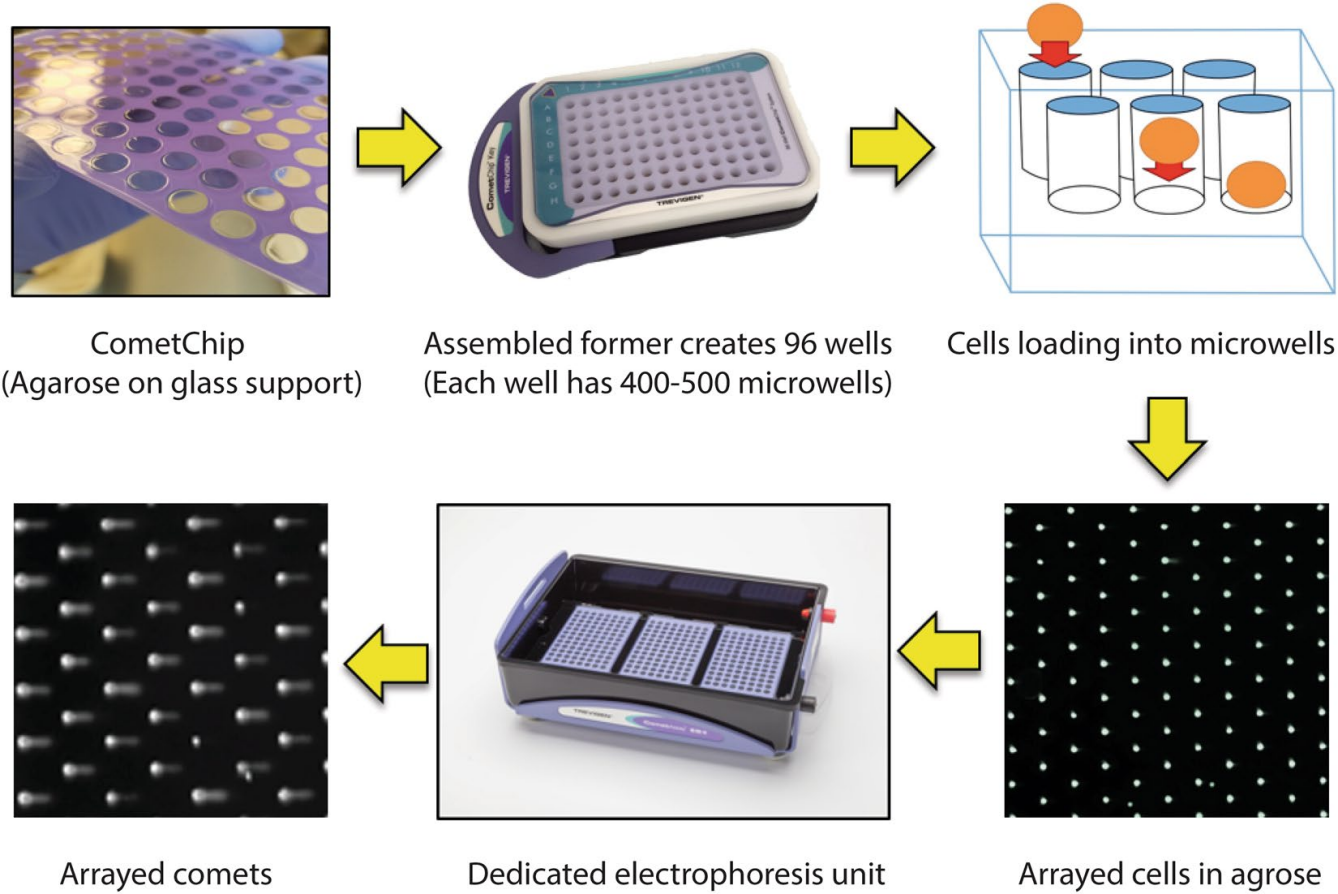
Design of Next-Generation CometChip hardware and work-flow. Close up of the CometChip showing the agarose surface chemically bound on a glass support. The CometChip is inserted into a Former base. When assembled, the Former allows each of the 96 wells in the CometChip to be treated individually. Each well has, in turn, more than 500 microwells that enable cells to be gravity loaded into the CometChip, the result is that all cells are on a single focal plane. The dedicated electrophoresis system limits the field variability by reducing the distance between the two electrodes. After electrophoresis, the CometChip is stained and images are acquired using an automated image cytometer (Celigo). These images are subsequently analyzed using dedicated CAS optimized for the CometChip high throughput methodology.

The underside of the glass slide that does not contact the agarose is stenciled in the pattern of a 96-well plate to provide reference for the user (Fig. 1). In the original design^21^, microwell arrays were created using a photolithography method. In short, a photo-resist SU 8000 template was used to create a dimethylsiloxane (PDMS) mold with micro-patterned pillars. This process required a step in which the mold was floated on molten agarose, which sometimes resulted in variation in thickness. To overcome this problem, we developed micro-patterned vertical casting cassettes that greatly improved the uniformity of the thickness of the molded agarose. The use of vertical casting cassettes is compatable with the creation of  defined microwell sizes (e.g., 20, 30, or 40 microns and a depth of approximately 50 microns) to accommodate most cell types. Furthermore, the cassettes can be designed to create single chips that have a variety of microwell sizes. The Multisize-CometChip contains 4 rows each of microwells with sizes of 20, 30, or 40 microns. The Multisize-CometChip can be used to select the appropriate microwell diameter for the cells under study and to examine whether cell diameter impacts the number of cells loaded into the microwell within a single experiment.

Another important advance was the creation of a novel macrowell former, which converts the CometChip into a 96-well format compatible with multi-channel pipettes (Fig. 1, Assembled former). The macrowell former consists of 96 bottomless wells arranged in the same dimensions of a standard 96-well plate and four magnets on the underside that are in-line with the partnered magnets on the base. Assembling the macrowell former and the base containing the CometChip results in 96 wells, each able to hold approximately 250 μl of liquid with an agarose floor containing approximately 500 microwells. In addition, this system contains a key to unlock the assembled system to allow removal of the CometChip after cell loading and treatment. As previously described, cells can be first loaded into the system and then exposed (e.g. radiation, chemicals, complex mixtures, etc.), or, alternatively, cells can be treated in traditional 96-well plates and subsequently loaded into the CometChip^21^.

### The Comet Electrophoresis System (CES)

The CES is an electrophoresis unit designed to run up to three CometChips simultaneously (Fig. 1, electrophoresis unit). In contrast to most rectangular horizontal electrophoresis systems, electrophoresis in the CES is performed along the short axis of the unit. We had found that when electrophoresis units are run using alkaline conditions, the electrical field is non-homogenous. However, electrophoresis run along the shorter axis of the apparatus minimizes the distance between electrodes and considerably improves the homogeneity of the electric field, eliminating unacceptable well-to-well inconsistencies when electrophoresis is performed under alkaline conditions. In the CES, the CometChips are placed in a tray designed to maintain the correct orientation during electrophoresis.

### Image acquisition and Comet Analysis Software (CAS)

**Figure 2:**
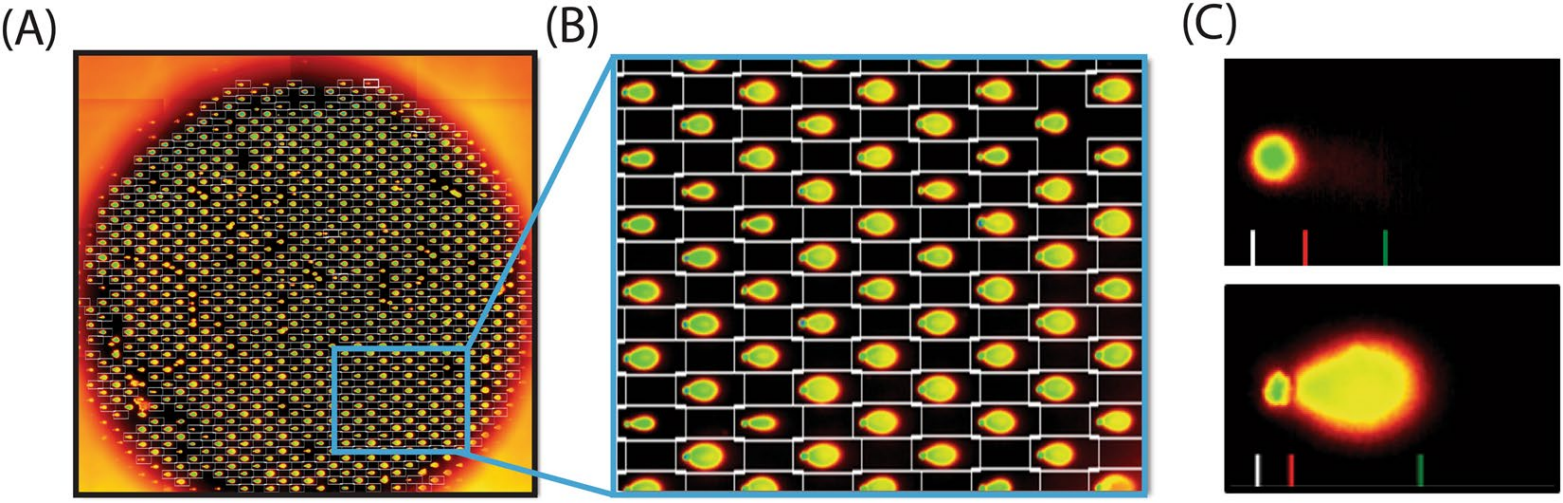
Acquisition and analysis of image data. (**A**) Image acquisition and comet analysis using dedicated CAS. Representative image of a single CometChip well after DNA damage treatment. Image is comprised of 16 individual post-acquisition stitched images. The insert shows the region expanded in (**B**). (**B**) Higher magnification of the insert from (**A**), shows individual wells. The CAS automated analysis software detects and boxes each comet and colors the comet based on the intensity of the signal. (**C**) Two examples of comets post-CAS analysis – (**upper**) negative control comet with endogenous level of DNA damage. The DNA does not migrate significantly due to supercoiling. (**lower**) Example of a comet from a highly-damaged cell that has been exposed to etoposide. In each example, the white line at the bottom of the image marks the beginning of the comet head, the red line marks the outermost edge of the comet head and the green line marks the end of the comet tail.

Imaging was conducted using the Celigo S Imaging Cytometer (controller program V3.0.3.2), allowing rapid, high-resolution data acquisition. For the research described here, all images were taken at 1 micrometer/pixel in the 96-well plate setting. The Celigo S captures 16 images of each well that are then stitched into a single representation (Fig. 2A). These images were then uploaded into the CAS. The dedicated CAS automatically locates comets within images and calculates measurements of DNA damage. It uses a multi-dimensional classification model to distinguish scorable comets from other objects found in comet assay images. The individual analytic dimensions of the classifier model derive from characterizing measures acquired on objects found in the images, by the systematic application of a variety of mathematical image processing techniques. These techniques seek first to partition objects of potential interest from the background using a scanning box coupled with an adaptive thresholding routine (Fig. 2B). They then derive several quantitative measures, which characterize the found objects in terms of the various dimensions of the classifier model. Quantitative limits related to each analytic dimension then allow the finding routines to classify a candidate object as either a comet or a non-target object. While the total process is complex, the underlying classification is conceptually simple. The CAS answers questions such as:

- Is this object too small to be a comet?
- Is it too big?
- Is the orientation of the long axis of the object consistent with the direction of electrophoresis?
- Is the object’s shape consistent with the expected shape of a comet?
- Is the object symmetrical about a horizontal line drawn through its center?

By asking such questions, the CAS systematically sieves through the objects found on each image and classifies them as potentially scorable comets, or as non-target objects to be rejected from further analysis. The objects judged to be consistent with the characteristics of comets are then passed on to additional software routines, which analyze and quantify the associated distribution of fluorescent intensities and derive various measures of DNA damage (Fig. 2C). These additional routines first fit an interpolated surface to the background intensities surrounding a comet to model the local pattern of non-specific background fluorescence. This fitted surface is then subtracted from the comet, leaving a corrected pixel intensity distribution that solely reflects the comet’s DNA distribution. Strategic profiles and projections of the corrected intensity distribution are then generated and analyzed to identify the most likely position of the dividing line between the head and tail region (if any) of the comet (seen as a red line, Fig. 2C), as well as the location of the terminal end of the tail (green line, Fig. 2C). These regional markers are then used to guide the straightforward computation of various measures of DNA damage, such as tail length, percent DNA in the tail (% tail DNA) and tail moment from the background corrected intensity distribution (for addition details, see Supplemental text).

### Validation of the CAS algorithm

Simulated comet images (Fig. S1B) were designed to represent a range of damage levels and were constructed by using circular and elliptical shapes of selected intensity (grey scale) values to mimic the shape and intensities of typical comets. These comet images were de-convoluted with a 15 × 15 smoothing filter to make the appearance of the simulated comets more realistic. These images were combined into a single image and arranged such that their order of processing by the program was predictable. Values for the % tail DNA, tail moment, and tail length were manually calculated during the simulation process. Simulated images were then scanned using the CAS, and % tail DNA values plotted against the manually calculated value. As can be seen in Fig. S1C, there is a strong linear relationship between the calculated values and those obtained with the CAS (*r*^2^ > 0.98). It has also been established that there is a strong linear relationship between comets of the CometChip and those of the traditional slide-based comet assay when evaluated using the Loats Automated Comet Assay Scoring System (*r*^2^ > 0.99) (Fig. S1D). This is consistent with that observed for the initial cometchip methodology, the spatially encoded microwell comet assay, showing a linear dose response (upon exposure to ionizing radiation) when comparing the microwell comet assay and the traditional comet assay^21^.

### Assessment of CometChip loading

After validation of the CAS, we evaluated the loading parameters of the CometChip. Considering that the average cell size used in this study varies between 15 and 25 microns, there remained the possibility that more than one cell could be loaded into a single well. This was evaluated using two isogenic cell lines expressing different fluorescent protein markers^21^, developed as previously described^27,28^. The experiment was conducted using the Multisize-CometChip, that is comprised of microwells of 20, 30 and 40 microns, and with MDA-MB-231/GFP and MDA-MB-231/RFP cells, that have an average diameter of 20 microns. As anticipated, the cell size prevented the cells from settling into the 20-micron wells; consequently, the wells loaded poorly (<20% loading), confirming that efficient loading requires microwells be slightly larger than the cells of interest (Fig. S1E,F). However, the 20 micron microwells had less than 1% multiple-loading of cells when a concentration of 10^5^ cells/ml was used. As microwell size increased, the percentage of multiple-loaded microwells increased proportionally. With 30-micron wells, approximately 12–18% had multiple cell loading. Increasing microwell size to 40 microns (twice the average cell diameter) resulted in multiple cell loading in 30–36% of the microwells. We suggest users initially measure the diameter of the target cells or empirically evaluate loading using the Multisize-CometChip that is comprised of microwells at 20, 30 and 40 microns. Such an initial test run would ensure efficient loading for the cell type under study and would help minimize multiple cell loading. We also suggest using 2–3 wells per chemical dose and multiple runs when generating dose-response data to ensure adequate statistical power. In many of the runs shown here, this approach provided >1000 comets per data point, markedly reducing error and increasing statistical significance.

### CometChip intra- and inter-assay variability

**Figure 3:**
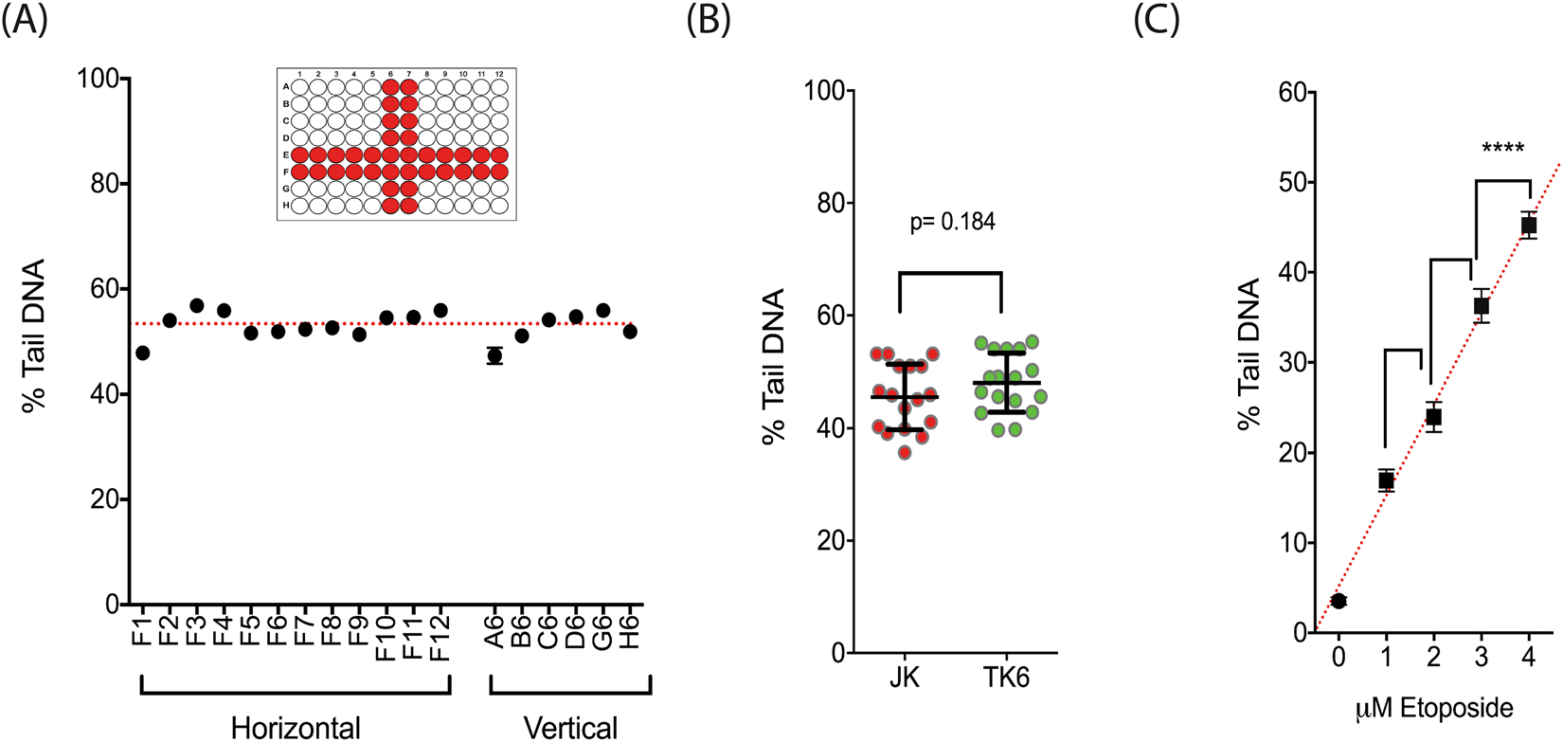
CometChip validation. (**A**) Intra-assay variability. The wells on the outer edge of the plate had the most variability compared to controls. Overall, we observed average variability of less than 4.2% of mean difference and maximum variability less than 10% of mean difference. The average group value is depicted as a red dotted line. Error bars = mean with 95% CI (n = 644–749). (**B**) Inter-assay variability. Cells were treated with etoposide (5 μM) and DNA damage was measured after 1 h. Each point represents a separate experiment (n = 17 per cell line). CV was measured at 13% for the JK cells and 11% for the TK6 cells (n = 420–580 per point). Error bars = mean ± SD. Statistical analysis was conducted via a two-sided Student’s t-test. (**C**) Linear DNA damage response to the DSB causing agent, etoposide. Assay was able to resolve differences in DNA damage caused by 1 μM increments of the compound (****p < 0.0001, n = 1000–2000 comets; *r* = 0.98). Statistical analysis was conducted via a two-sided Student’s t-test.

A recognized limitation of the standard comet assay is intra/inter-assay reproducibility. To determine the amount of intra-assay variability across the CometChip platform, Jurkat cells were treated with the genotoxic agent etoposide (5 µM, 1 h) and measured for DNA damage (Fig. 3A). The treatment yielded a damage average of 52.8% tail DNA going horizontally across the plate (Row E, F) and 53.3% tail DNA in wells going vertically down the plate (Column 6,7). Maximum deviation from mean occurred in the peripheral wells (those on the outer most edge of the plate) reaching a value of ±5.5% mean value, whereas deviation from the mean in the internal wells was substantially lower, at ± 2.34% mean value (Table S1). These results confirm that the CometChip Platform described herein has low intra-assay variability that is similar to or better than the original cometchip^21^, and calls attention to increased variation along the edges (edge effects have been observed in many contexts).

To measure inter-assay variability for the CometChip, we compared results between multiple assays conducted within our laboratory over a three-month time period. An internal 5 μM etoposide positive control was included in many of these CometChip assays. This internal control was subsequently used to measure the amount of inter-assay variability. Thirty-four assays, seventeen each for the Jurkat and TK6 cell lines, were conducted by three researchers (20, 8, and 6 assays per individual) (Fig. 3B). The CometChip platform yielded reproducible results for the etoposide internal positive control among experiments (Table S2). Average difference from mean was less than 10% across all assays. For Jurkat and TK6 cells, CV was calculated at 13% and 11%, respectively. These data confirm that the CometChip Platform has less inter- and intra-assay variability than the slide-based comet assay. Research groups will need to validate this system within their own laboratory and we suggest using these data as a standard for that validation.

### CometChip sensitivity

Improvements in both the CometChip hardware design and image analysis allowed for precise measurements of DNA damage. To evaluate precision, Jurkat cells were exposed to concentrations of etoposide ranging from 1–4 μM. The assay was able to distinguish differences in DNA damage levels at 1 μM increments of etoposide and did so with a high degree of confidence (p < 0.0001) (Fig. 3C). This experiment was also duplicated in an adherent human cell line (HCT116) using a wider range of etoposide concentrations (Fig. S1G,H). Interestingly, HCT116 cells showed minimal DNA damage at the 5 µM dose, requiring > 35 µM to achieve over 50% tail DNA. The same was found in the HCT116/p53-KO cells.

### Analysis of DNA damaging agents

**Figure 4:**
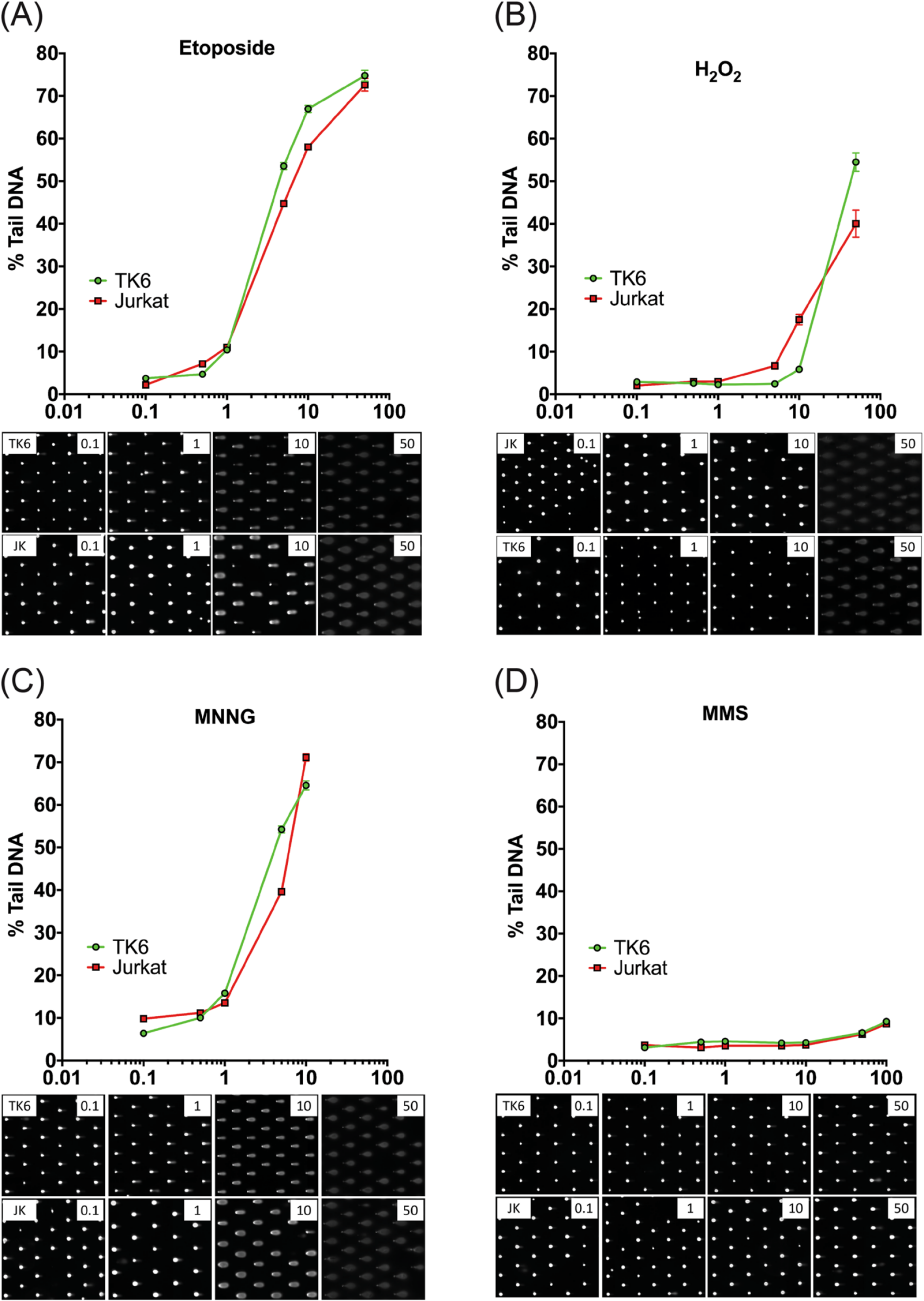
Analysis of known DNA damaging agents. (**A**) Etoposide is shown to induce replication-dependent DSBs and cause significant DNA damage at concentrations greater than 1 μM (n = 1069–1374). Shown is the plot of % tail DNA for TK6 and Jurkat cells following exposure (60 min) to the agent at the doses indicated. (**B**) H_2_O_2_ did not cause DNA damage below 1 μM, with elevated levels of DNA damage observed at 50 μM (n = 450–1200). Shown is the plot of % tail DNA for TK6 and Jurkat cells following exposure (60 min) to the agent at the doses indicated. (**C**) MNNG, a potent SN1 DNA alkylating agent^67^, was the most genotoxic of the compounds tested and caused the maximum amount of DNA damage that could be measured at 10 μM (n = 1075–1374). Shown is the plot of % tail DNA for TK6 and Jurkat cells following exposure (60 min) to the agent at the doses indicated. (**D**) MMS, an SN2 DNA alkylating agent^67^, induced only modest DNA damage on the cells tested, with significant DNA damage observed only at concentrations above 50 μM (n = 812–1294). All assays were conducted in duplicate. Error bars represent mean ± 95% CI. Shown is the plot of % tail DNA for TK6 and Jurkat cells following exposure (60 min) to the agent at the doses indicated. For each panel, the images below the plot are representative CometChip images at the doses indicated.

As a measure of CometChip capability, we tested four well-documented DNA damaging agents representing different modes of action: etoposide (a topoisomerase II poison^29^), hydrogen peroxide (H_2_O_2_), MNNG (SN1 alkylating agent), and MMS (SN2 alkylating agent) (Fig. 4A–D). Etoposide was tested in TK6 and Jurkat cells using a wider range of doses (0.1–100 μM) than were used in the previous experiments. Etoposide induced high levels of DNA damage (~40% tail DNA) at concentrations as low as 5 μM (Fig. 4A). In contrast to etoposide, H_2_O_2,_ which induces a variety of DNA damage types including oxidative lesions and single-stranded breaks (SSB)^30^, induced high levels of DNA damage only at concentrations higher than 10 μM (Fig. 4B). MNNG was shown to be a potent inducer of DNA damage, as expected, since human lymphocytes have been reported to be particularly sensitive to alkylating agents; at concentrations above 10 μM, the amount of MNNG-induced damage exceeded the capacity for measurement (Fig. 4C). In contrast, when the cells were exposed to 100 µM MMS, another alkylating agent, no damage was detected after a short 30 min exposure (Table S3). Detectable DNA damage occurred at a concentration of 100 µM MMS only after a 60 min exposure time (Fig. 4D).

### Endogenous DNA damage and measurement of DNA repair kinetics

An advantage of the CometChip Platform, compared to slide-based comet protocols, is the ability to analyze thousands of comets under identical experimental conditions. This development makes it possible to quickly measure parameters that were ill-suited to slide-based comet analyses, including measuring endogenous levels of DNA damage and the kinetics of DNA repair after treatment. To that end, we used a human colon tumor-derived cell line (HCT116) with a CRISPR/Cas9-mediated mutation in the first exon of *POLB* to test whether knockout (KO) of this DNA repair gene influenced the level of DNA damage in HCT116 cells, with or without previous exposure to genotoxicants. Two independently-developed HCT116/Polβ^−/−^ cell lines were created (HCT116/Polβ^sg1.3^ and HCT116/Polβ^sg1.5^) using alternate gRNA constructs targeting the first exon.

**Figure 5:**
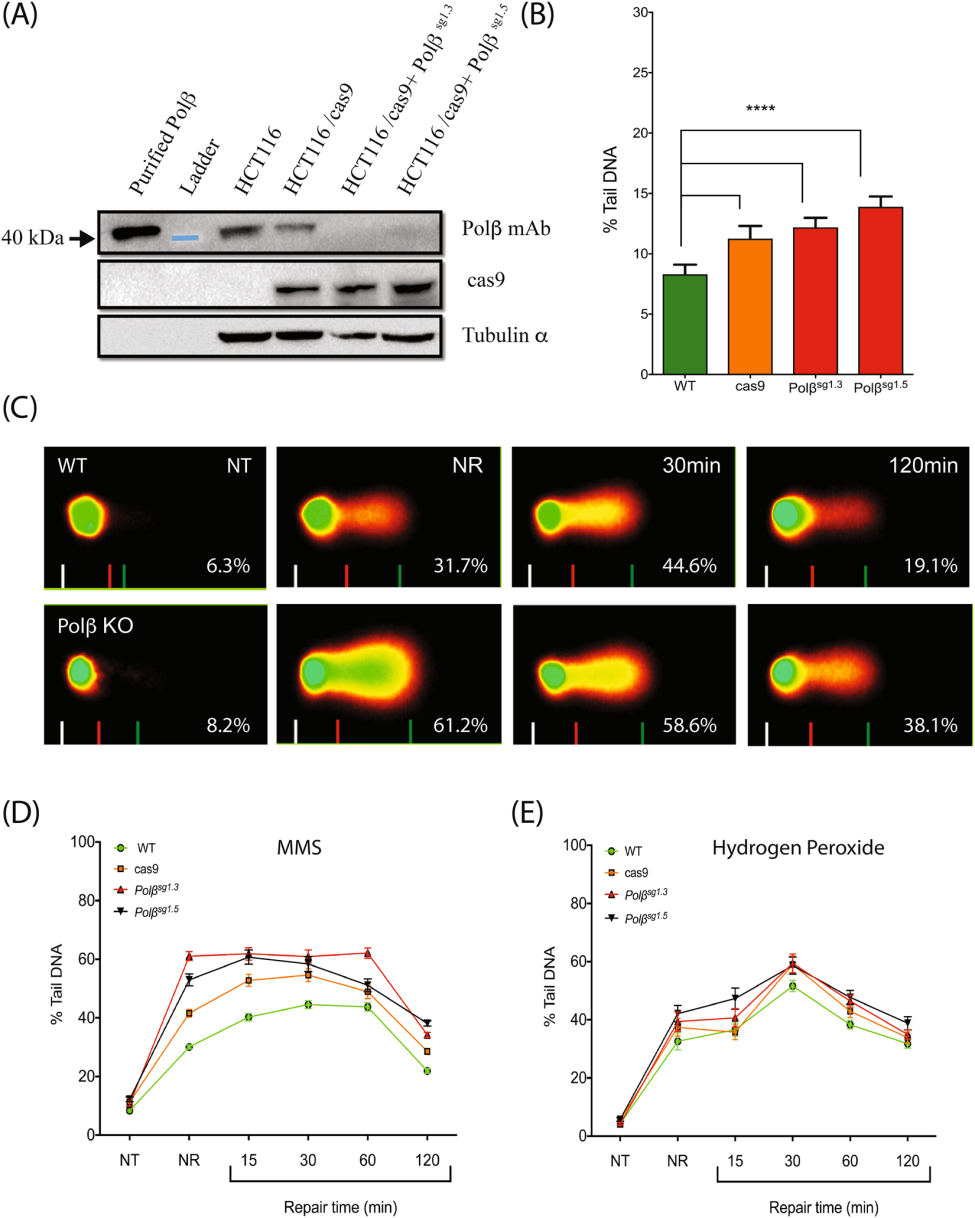
Endogenous DNA damage measurements and repair of DNA damage. (**A**) Validation of CRISPR-mediated KO of Polβ (gRNA 1.3/1.5) in HCT116 cells by immunoblot. Whole cell extracts were probed for Polβ at D12 post transduction to confirm KO and also for the expression of Cas9. Tubulin-α was used as a loading control. Blue band represents the 40 kDa marker visible on the membrane and the position of the 40 kDa molecular weight marker is indicated on the left. (**B**) Comparison of endogenous DNA damage levels. Cas9 containing cells had significantly higher levels of damage compared to the parental (WT) line (****p < 0.0001). All assays were conducted at least in duplicate. Statistical analysis was conducted via one-way ANOVA. (**C**) Representative images of CAS output from WT and Polβ-KO^g1.3^ after treatment with the DNA alkylator MMS (1 mM, 60 min): NR = no repair, NT = no treatment, 30 min and 120 min are durations of repair. Percentages are % tail DNA. (**D**) Repair kinetics of Polβ deficient cells after treatment with MMS (1 mM) or (**E**) H_2_O_2_ (50 μM). Error bars are mean ± 95% CI. Experiments conducted in duplicate.

The expression of Polβ protein was analyzed in these two Polβ-KO cell lines, and compared to a negative control containing Cas9 and an empty gRNA plasmid (Cas9) and compared to the original unaltered parental cell line (WT). The CRISPR-mediated gene modification resulted in undetectable levels of Polβ protein as determined by immunoblot analysis (Fig. 5A). Evaluating the levels of endogenous DNA damage in the various cells showed that damage levels were comparable across the three altered cell lines (HCT116/Cas9, HCT116/Polβ^sg1.3^, and HCT116/Polβ^*s*g1.5^), and all lines had significantly higher levels of DNA damage than the untransformed parental line (WT) (Fig. 5B). These results suggest that constitutive Cas9 expression may cause elevated levels of DNA damage irrespective of the levels of Polβ in the cell. To test the effect of altered Polβ levels on DNA repair capacity, the cells were exposed to 1 mM MMS (60 min), to which Polβ deficient cells are known to be sensitive^31,32^. Cells deficient in Polβ accumulated approximately 2- to 3-fold more DNA damage after the treatment compared with WT cells. However, comparison of inter-cell line repair kinetics did not suggest that the Polβ deficient cells repaired the accumulated DNA damage any slower (Fig. 5C and D). We also evaluated the repair kinetics of the cells after H_2_O_2_ treatment. Hydrogen peroxide induces oxidative DNA damage at low concentrations and Polβ is active in base excision repair (BER)^33^, a repair pathway invoked in response to oxidative damage. As observed with MMS, the transformed cell lines accumulate a higher level of initial DNA damage but repaired the damage at a rate comparable to Cas9 cells (Fig. 5E).

### Patient derived lymphocytes

**Figure 6:**
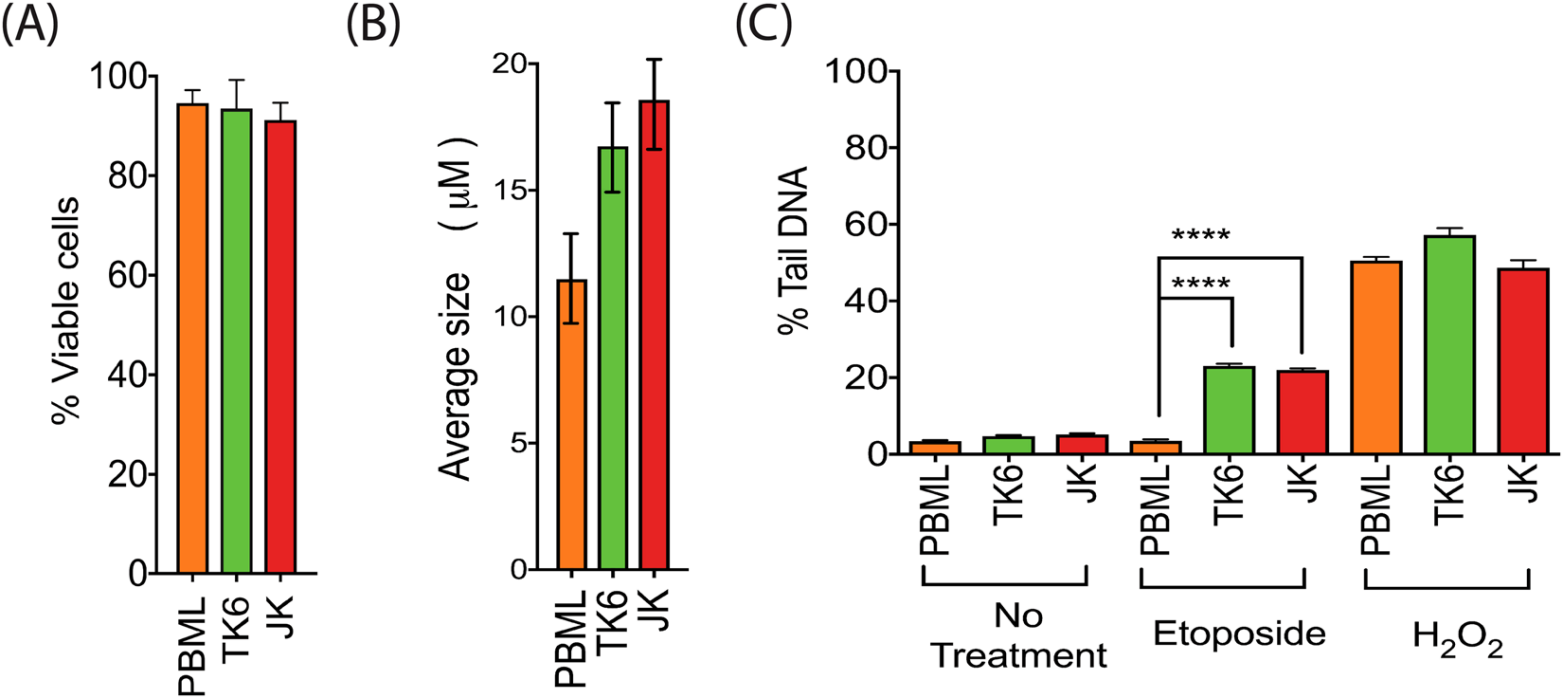
Endogenous and induced DNA damage measured in patient derived lymphocytes. (**A**) Average cell size. Patient derived cells (peripheral blood mononuclear lymphocytes, PBMLs) were smaller that the two cultured cell lines (TK6/JK). Data shows the mean of more than 100 cells. (**B**) Cell viability remained high after the lymphocytes were separated from the whole blood. Measured by trypan blue exclusion. (**C**) Levels of endogenous DNA damage was comparable between the three groups (n = 2400–2500). When exposed to etoposide (5 μM, 30 min exposure), only the replicating cells acquired DNA damage (n = 1900–2841). Conversely, all cell types acquired comparable amounts of DNA damage after exposure to H_2_O_2_ (50 μM, 30 min exposure). Statistical analysis was conducted via a two-sided Student’s t-test.

We have shown that one of the strengths of the CometChip Platform is the ability to assay multiple samples simultaneously, which has direct application to studies that require sampling large patient cohorts. To test this application, we collected primary lymphocytes from a donor and separated them from whole blood by centrifugation using BD vacutainer CPT™ tubes; this process did not compromise the viability of the lymphocytes (Fig. 6A). Prior to CometChip loading, the size of the primary lymphocytes was then measured and found to be smaller as compared to the two human-derived immortalized lymphocyte lines (TK6 and Jurkat, JK) (Fig. 6B). We then compared endogenous levels of DNA damage between the primary cells and the two cell lines under the same conditions using the CometChip protocol (Fig. 6C) and found that the level of endogenous damage was comparable among the three cell types, despite their genetic variability. We also measured the effect of etoposide, an agent that preferentially affects proliferating cells (Fig. 6C)^34^. Etoposide (5 μM, 1 h exposure) had no detectable effect on the primary lymphocytes while significantly (p < 0.0001) increasing DNA damage levels in the proliferating TK6 and Jurkat cells, confirming the resistance of non-dividing cells to etoposide. To confirm that the patient-derived lymphocytes could respond to DNA damaging agents in a manner measurable by CometChip analysis, the cells were also exposed to H_2_O_2_ (50 μM, 1 h exposure). The primary lymphocytes and the two cell lines showed comparable levels of DNA damage following H_2_O_2_ treatment (Fig. 6C). These results confirm that freshly isolated primary human lymphocytes can be used in the CometChip, thus setting the stage for this CometChip Platform to be used in human population studies.

### NTP Compound Screen

**Figure 7:**
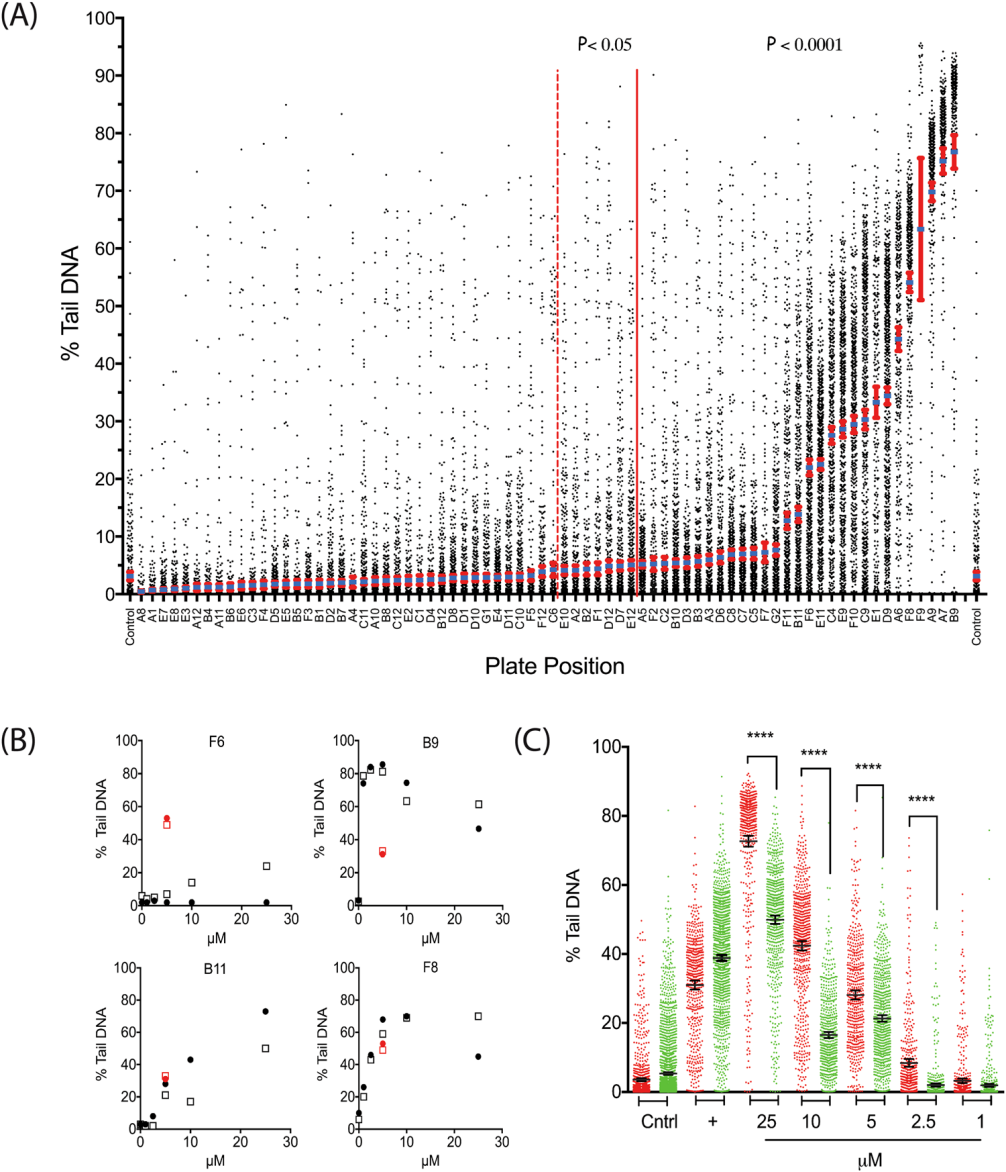
NTP compound plate screen. (**A**) Prescreen data from all compounds (A1–G2), showing all points and spread of data. Error bars represent mean +/− 95% CI. Also refer to Tables 1 and 2. Statistical analysis was conducted via a one-sided students t-test. (**B**) Representative graphs from four compounds. Compound B9 (Thiram) was one of the most genotoxic compounds tested. Cells exposed to compound F8 (another dithiocarbamate compound) also had high levels of DNA damage at low concentrations. Compounds F6 and B11 showed differential response based on p53 status of the cells. Symbols: squares = TK6 and circles = Jurkat (JK). Red symbols are etoposide internal controls for each of the cell lines. (**C**) Dose-response analysis of compound B11 shows that the JK cells (red) are slightly less sensitive to etoposide (+) control than the TK6 cells (green). Despite this, the JK cells show higher levels of DNA damage than the TK6 cells following treatment with compound B11 (****p < 0.0001). Statistical analysis was conducted via a two-sided Student’s t-test.

Because greatly enhanced throughput is one of the key advancements of the CometChip Platform, we investigated whether it could be used to screen a large collection of environmental compounds to evaluate their potential as DNA damaging agents. To that end, we evaluated a plate of 74 compounds selected by and sourced from the NTP (Tables 1, 2) that contained compounds in four categories: known direct-acting genotoxicants (based on standard genetic toxicity tests, e.g., MNNG), known non-genotoxic compounds (e.g., benzyl alcohol), possible genotoxicants, (compounds that were active in Tox21 quantitative high throughput screening (qHTS) assays for DNA damage but with no data from standard genetic toxicity tests (e.g., tribromoacetaldehyde), and true unknowns (compounds with no genetic toxicity or Tox21 assay data (e.g., bisphenol E)^35–40^. For most compounds, requirements for metabolic activation were also known. The known genotoxicants were chosen to cover a variety of modes of action, as well as different activity profiles in the Tox21 high-throughput DNA damage screens (Table S3). Our preliminary CometChip screen was conducted with Jurkat cells using an acute exposure (30 min) at a single concentration (100 µM). Of the 27 ‘known genotoxicants’ obtained from the NTP (Table 3), 12 showed significant increases in DNA damage compared to the control (11 at p < 0.0001 and 1 at p < 0.05). Of the 28 ‘possible genotoxicants’, 15 showed significant increases in DNA damage compared to the control (13 at p < 0.0001 and 2 at p < 0.05 at the 100 µM dose used). Of the 17 ‘known non-genotoxicants’, 6 showed significant increases in DNA damage compared to the control (4 at p < 0.0001 — melamine, curcumin, cyclohexanone and progesterone, and 2 at p < 0.05 — phthalic anhydride and di-(2-ethylhexyl) phthalate). Of the 2 true unknowns, neither podofilox nor bisphenol E induced DNA damage at 100 µM, 30 min exposure (Table 2). Overall, analysis of the CometChip screen outcome data using a one-sided t-test revealed a total of 28 genotoxicants at the p < 0.0001 level and 6 at the p < 0.05 level (Table S4). To ensure that the mean value was an accurate representation of the data, we generated dot-plots to identify potential sub-populations that could skew the results (Figs 7A and S2A–G).

**Table 1:** National Toxicology Program 74 compound plate – agents scored as damaging by CometChip (in descending order by mean % tail DNA within each category). (P value was calculated using a one tailed Student’s t-test).

Chemical Name	CAS #	Plate Position	*A priori* designation	P Value
Thiram	137-26-8	**B9**	Known genotoxicant	P < 0.0001
Hydroquinone	123-31-9	**A9**	Known genotoxicant	P < 0.0001
2-Aminoanthraquinone	117-79-3	**D9**	Known genotoxicant	P < 0.0001
Cadmium chloride	10108-64-2	**C4**	Known genotoxicant	P < 0.0001
D&C Yellow 11	8003-22-3	**F11**	Known genotoxicant	P < 0.0001
N-Methyl-N’-nitro-N-nitrosoguanidine (MNNG)	70-25-7	**G2**	Known genotoxicant	P < 0.0001
Sodium dichromate(VI) dihydrate	7789-12-0	**C5**	Known genotoxicant	P < 0.0001
Styrene oxide	96-09-3	**C7**	Known genotoxicant	P < 0.0001
2,4-diaminotoluene	95-80-7	**C8**	Known genotoxicant	P < 0.0001
Adriamycin HCl (a.k.a. Doxorubicin HCl)	25316-40-9	**A3**	Known genotoxicant	P < 0.0001
1-Ethyl-1-nitrosourea (ENU)	759-73-9	**C2**	Known genotoxicant	P < 0.0001
Diethylstilbestrol	56-53-1	**D7**	Known genotoxicant	P < 0.05
Trichloroethylene	79-01-6	**A2**	Known genotoxicant	P < 0.05
Malachite green oxalate	2437-29-8	**A7**	Possible genotoxicant	P < 0.0001
Tribromoacetaldehyde	115-17-3	**F9**	Possible genotoxicant	P < 0.0001
Copper dimethyldithiocarbamate	137-29-1	**F8**	Possible genotoxicant	P < 0.0001
Zinc dibutyldithiocarbamate	136-23-2	**A6**	Possible genotoxicant	P < 0.0001
2,4-Di-tert-butylphenol	96-76-4	**E1**	Possible genotoxicant	P < 0.0001
Poly(oxy-1,2-ethanediyl),.alpha.-hydro-.omega.-[(1-oxo-2-propenyl)oxy]-, ether with 2-ethyl-2-(hydroxymethyl)-1,3-propanediol (3:1)	28961-43-5	**F10**	Possible genotoxicant	P < 0.0001
3-Butyl-1-nitro-3-nitrosoguanidine	13010-08-7	**E11**	Possible genotoxicant	P < 0.0001
1-Methyl-3-tetradecylimidazolium chloride	171058-21-2	**F6**	Possible genotoxicant	P < 0.0001
2-(2H-Benzotriazol-2-yl)-4-methylphenol	2440-22-4	**B11**	Possible genotoxicant	P < 0.0001
1-Methyl-3-tetradecylimidazolium bis(trifluoromethylsulfonyl)imide	404001-49-6	**F7**	Possible genotoxicant	P < 0.0001
Bisphenol AF	1478-61-1	**D6**	Possible genotoxicant	P < 0.0001
p-(2,3-Epoxypropoxy)-N,N-bis(2,3-epoxypropyl)aniline	5026-74-4	**D3**	Possible genotoxicant	P < 0.0001
4-Aminoazobenzene	60-09-3	**B10**	Possible genotoxicant	P < 0.0001
6-Azacytidine	3131-60-0	**E12**	Possible genotoxicant	P < 0.05
3’-Methyl-4-dimethylaminoazobenzene	55-80-1	**D12**	Possible genotoxicant	P < 0.05
Cyclohexanone	108-94-1	**C9**	Non-genotoxicant	P < 0.0001
Melamine	108-78-1	**E9**	Non-genotoxicant	P < 0.0001
Progesterone	57-83-0	**B3**	Non-genotoxicant	P < 0.0001
Curcumin	458-37-7	**A5**	Non-genotoxicant	P < 0.0001
Di-(2-ethylhexyl)phthalate	117-81-7	**B2**	Non-genotoxicant	P < 0.05
Phthalic anhydride	85-44-9	**E10**	Non-genotoxicant	P < 0.05

**Table 2:** National Toxicology Program 74 compound plate – agents scored as non-damaging by CometChip (in descending order by mean % tail DNA within each category).

Chemical Name	CAS #	Plate Position	*A priori* designation
Glycidol	556-52-5	**C6**	Known genotoxicant
Diglycidyl resorcinol ether	101-90-6	**E4**	Known genotoxicant
4-(Dimethylamino)azobenzene	60-11-7	**G1**	Known genotoxicant
1-Amino-2-methylanthraquinone	82-28-0	**D8**	Known genotoxicant
Colchicine	64-86-8	**C1**	Known genotoxicant
Bisphenol A	80-05-7	**B8**	Known genotoxicant
Azidothymidine	30516-87-1	**A10**	Known genotoxicant
17beta-Estradiol	50-28-2	**B7**	Known genotoxicant
2-Acetylaminofluorene	53-96-3	**E5**	Known genotoxicant
Black cohosh extract	84776-26-1	**F4**	Known genotoxicant
Methyl Methanesulfonate (MMS)	66-27-3	**C3**	Known genotoxicant
6-thioguanine	154-42-7	**A11**	Known genotoxicant
Ethyl methanesulfonate (EMS)	62-50-0	**E3**	Known genotoxicant
Cisplatin	15663-27-1	**A1**	Known genotoxicant
4,4'-Thiodiphenol	2664-63-3	**F2**	Possible genotoxicant
Selenium oxide	7446-08-4	**F1**	Possible genotoxicant
4-Azoxyanisole	1562-94-3	**F12**	Possible genotoxicant
Daidzein	486-66-8	**D11**	Possible genotoxicant
Phenethyl anthranilate	133-18-6	**D10**	Possible genotoxicant
Lauryl gallate	1166-52-5	**D1**	Possible genotoxicant
1,3-Diiminobenz(f)isoindoline	65558-69-2	**B12**	Possible genotoxicant
2,4,4'-Trihydroxybenzophenone	1470-79-7	**D4**	Possible genotoxicant
N-Phenyl-1-naphthylamine	90-30-2	**E2**	Possible genotoxicant
2,2',5,5'-Tetrachlorobenzidine	15721-02-5	**D2**	Possible genotoxicant
2-Chloroethyldiethylammonium chloride	869-24-9	**F3**	Possible genotoxicant
Tetraphenylolethane glycidyl ether	7328-97-4	**D5**	Possible genotoxicant
Digoxin	20830-75-5	**B6**	Possible genotoxicant
N.N’-dicyclohexylthiourea	1212-29-9	**C10**	Non-genotoxicant
Tris(2-ethylhexyl)phosphate	78-42-2	**C12**	Non-genotoxicant
Fluometron	2164-17-2	**C11**	Non-genotoxicant
n-Butyl Chloride (a.k.a. 1-Chlorobutane)	109-69-3	**A4**	Non-genotoxicant
Phenformin HCl	834-28-6	**B1**	Non-genotoxicant
Benzyl alcohol	100-51-6	**B5**	Non-genotoxicant
Ampicillin trihydrate	7177-48-2	**E6**	Non-genotoxicant
Urea	57-13-6	**B4**	Non-genotoxicant
D-Mannitol	69-65-8	**A12**	Non-genotoxicant
D-Limonene	5989-27-5	**E8**	Non-genotoxicant
(2-Chloroethyl)trimethyl-ammonium chloride (Chlormequat chloride)	999-81-5	**E7**	Non-genotoxicant
Bisphenol E	2081-08-5	**F5**	True unknown
Podofilox	518-28-5	**A8**	True unknown

**Table 3:** 27 known genotoxic agents in the NTP plate and results of the CometChip pre-screen (100 µM concentration, 30-min exposure duration).

Chemical Name^a^	Rationale for lack of response in CometChip assay	Expected to induce DNA damage
Adriamycin HCl	n/a	Yes
Hydroquinone	n/a	Yes
Thiram	n/a	Yes
1-Ethyl-1-nitrosourea (ENU)	n/a	Yes
Cadmium chloride	n/a	Yes
Sodium dichromate (VI) dihydrate	n/a	Yes
Styrene oxide	n/a	Yes
2,4-diaminotoluene	n/a	Yes
Diethylstilbestrol	n/a	Yes
2-Aminoanthraquinone	n/a	Yes
D&C Yellow 11	n/a	Yes
N-Methyl-N’-nitro-N-nitrosoguanidine (MNNG)	n/a	Yes
Trichloroethylene	n/a	Yes
Glycidol	Reactive epoxide group, approached significance (*p* = *0.058*); inactive in all Tox21 assays suggesting requirement for conc. >100 µM (Table S3).	Yes
4-(Dimethylamino)azobenzene	Requires S9 for positive responses in NTP tests (MOLY, Ames)^c^; positive only in the Tox21 ATAD5 assay	Unclear
1-Amino-2-methylanthraquinone	Requires S9 for + response in NTP Ames and *in vitro* CA assays^c^. Active in Tox21 ATAD5 assay (Table S3).	Unclear
Bisphenol A	Negative in all NTP *in vitro/in vivo* assays^b^, ^c^ (Table S3). DNA damage not observed until cells are exposed at doses > 100 µM for 4 h^48^.	Unclear
6-thioguanine	Nucleoside analog, requires > 30 min exposure. In HCT116 cells, requires 24 h exposure to induce DNA strand breaks^43^.	No
Azidothymidine	Nucleoside analog, requires > 30 min exposure; in H9 cells, requires 24 h exposure^41^	No
Cisplatin	Crosslinking agent; reduced DNA tail length (p < 0.0001). Negative in comet assay data^49^.	No
Diglycidyl resorcinol ether	Suspected crosslinking agent (Table S3) and^40^; reduced % tail DNA (Figure S4, p < 0.0001).	No
Methyl methanesulfonate (MMS)	Requires conc. >100 µM and/or longer exposure; positive after 1 h in our studies (Fig. 4D). DNA damage detected by the comet assay if exposed for 4 h at conc. > 0.1 mM^45^.	No
Ethyl methanesulfonate (EMS)	Requires conc. >100 µM. DNA damage seen in comet assay with exposure of 4 h, at >2 mM^45^.	No
Colchicine	Known aneugen.	No
2-Acetylaminofluorene	Requires metabolism; inconsistent responses in comet assays^44^.	No
17beta-Estradiol	Cell type specificity, requires prolonged exposure and metabolism^46^; may induce chromosomal damage via an aneugenic mechanism^38^ Ames negative, rodent MN negative^c^.	No
Black cohosh extract	Positive in rodent MN assay, possible aneugen; negative in Tox21 assays (top conc. 100 µM)^42,43^ and Ames negative^44^^c^.	No

^a^Compounds in gray tested positive in the pre-screening.

^b^Ames, mouse lymphoma cell, and *in vitro* chromosomal damage assays; *in vivo* MN assay.

^c^Chemical Effects in Biological Systems (CEBS). Research Triangle Park, NC (USA): National Toxicology Program (NTP). 10.22427/NTP-DATA-1.

In total, the NTP compound library contains 27 compounds classified as genotoxic based on assays with a variety of different endpoints such as the Ames test (bacterial mutagenicity), induction of chromosome aberrations *in vitro*, or induction of micronuclei *in vivo* (structural damage or chromosome loss), indicating different modes of action for these compounds (Table S3)^35–49^. Thus, classifying an agent as genotoxic does not imply that the agent is necessarily a direct acting DNA damaging agent. These 27 compounds were selected with the expectation that several would not produce a response in the CometChip assay, based on their known modes of action or requirements for metabolic activation. Of these 27 genotoxicants, 13 were active in the acute, single-dose CometChip experiment (listed as ‘known genotoxicant’, Tables 1 and 3). We reviewed the 14 agents that were not active to clarify why these agents were not detected in the CometChip pre-screen (Table 3). As will be detailed in the Discussion section, these 14 chemicals cause genotoxic effects when exposed to cells through mechanisms that are not expected to induce DNA damage after an acute exposure and so would not be predicted to show a change in % tail DNA in the comet or CometChip assay (Table 3). However, to investigate the role of exposure duration on the results obtained in the pre-screen for these 14 compounds, we incubated TK6 cells for 24 h in the presence of each compound. To allow the detection of extremely small changes in DNA damage, we modified the electrophoresis conditions (increased the field strength) to enhance assay sensitivity. As shown in Fig. S4, despite the increased sensitivity for detecting DNA damage, no significant changes in % tail DNA were seen for 11 of the 14 test chemicals. Three chemicals (2-acetylaminofluoride, ethyl methanesulfonate, and 4-(dimethylamino)azobenzene) showed small but statistically significant increases in DNA damage. Interestingly, di-glycidyl resorcinol ether (DGRE) showed a marked reduction in DNA damage, a response consistent with a DNA crosslinking mode of action.

Twenty-two of the 28 compounds that induced DNA damage in the CometChip assay (p < 0.0001) were further assessed over a dose range of 1–25 μM (in both Jurkat and TK6 cells). This more comprehensive screen identified 10 compounds that induced damage DNA at lower, more physiologically relevant concentrations (Table 4, Fig. S3). A strong response was seen with thiram (compound B9), which showed high activity even at sub-micromolar concentrations (Fig. 7B). The amount of DNA damage measured in both the TK6 and Jurkat cells after thiram exposure was similar to the high levels of DNA damage observed after exposures to other di-thiolcarbamate-containing compounds in the screen, including zinc dibutyldithiocarbamate (compound A6) and copper dimethyldithiocarbamate (compound F8), suggesting that compounds within this class are particularly effective DNA damaging agents (Fig. 7B). Further, two compounds showed a differential response based on p53 status. The first, 1-methyl-3-tetradecylimidazolium chloride (compound F6), induced a greater response in the p53 normal TK6 cells, but only at concentrations ≥10 μM (Fig. 7B). The second compound, 2-(2H-benzotriazol-2-yl)-4-methylphenol (compound B11), a component of some sunscreens^50^, induced significantly more DNA damage in the p53 mutant Jurkat cells (red dots) as compared to the TK6 cells (green dots) (Fig. 7C). However, the internal control (etoposide) revealed that TK6 cells were slightly more sensitive to etoposide-induced DNA damage than the Jurkat cells. Hence, in this example, the internal control allowed for more confidence that the result was not an artifact of the assay run conditions.

**Table 4:** Compounds identified as active at concentrations >25 μM in the dose-response screen.

Plate Position	CAS #	Formula	Compound Name	Figure
A6	**136-23-2**	C1836N2S4Zn	Zinc dibutyldithiocarbamate	S3A
A7	**2437-29-8**	C52H54N4O12	Malachite green oxalate	S3A
A9	**123-31-9**	C6H6O2	Hydroquinone	S3A
B9	**137-26-8**	C6H12N2S4	Thiram	S3A, 7B
B11	**2440-22-4**	C13H11N3O	2-(2H-Benzotriazol-2-yl)-4-methylphenol	7B, 7C
C9	**108-94-1**	C6H10O	Cyclohexanone	S3C
F6	**171058-21-2**	C18H35ClN2	1-Methyl-3-tetradecylimidazolium chloride	7B
F7	**404001-49-6**	C20H35F6N3O4S2	1-Methyl-3-tetradecyl-1H-imidazol-3-ium bis[(trifluoromethyl)sulf onyl]azanide	S3E
F8	**137-29-1**	C6H12CuN2S4	Copper dimethyldithiocarbamate	S3E, 7B
F9	**115-17-3**	C2HBr3O	Tribromoacetaldehyde	S3E

The 32 compounds identified in the prescreen were retested using lower concentrations ranging from 25 μM to 1 μM in Jurkat cells. These ten compounds caused significant DNA damage at one or more of the concentrations tested (p < 0.0001).

## Discussion

Spontaneous as well as induced DNA damage, if not repaired, drives cancer^51^, aging^52^ and neurodegenerative disorders^53^, highlighting the biological significance of failing to effectively repair damage to the genome. The newly developed CometChip hardware and software platform presented here represents a significant leap from the traditional comet assay used to measure DNA damage, with far greater sensitivity and throughput. Herein we describe novel hardware and software technologies based on the single cell trapping microarray approach^21^. We demonstrate the efficacy, robustness, and throughput of the CometChip Platform through analysis of DNA damage in several contexts, including different damaging agents, CRISPR-induced DNA repair deficient cell lines, and analysis of peripheral blood mononuclear cells for use in evaluation of basal and induced DNA damage in human populations. Our results demonstrate the potential of the CometChip Platform for use in large-scale DNA damage and repair studies with multiple applications including basic research, drug discovery, toxicity testing, environmental toxicology, and human epidemiological studies. Additional studies to clearly establish the benefits and limitations of the technology in these various areas of application are ongoing.

A key advance over the original cometchip^21^ is the hardware for macrowell formation wherein a specially designed bottomless 96-well plate is magnetically compressed onto the agarose such that each well has within it an agarose surface with hundreds of microwells. This simplified macrowell former is far easier to handle compared to the original binder-clip approach, and allows the researcher to quickly and easily disassemble the system without damaging the agarose. In addition, novel chemistry performed on the surface of the glass enables the agarose to adhere to the glass, resulting in the entire agarose microwell array being supported by glass. Together with a vertical pouring approach, the agarose-glass design enables the creation of highly consistent gel thickness and a solid glass substrate that is easy to handle, features that improve the robustness of the assay.

In addition to improvements in the hardware, we have also created dedicated high-throughput CometChip analysis software (CAS). Although there are many available software packages for analysis of % tail DNA and related comet parameters, the time involved for analysis is still considerable. Direct evaluation and quantification by our group of the >40,000 comets on a single 96-well CometChip, using a stand-alone microscope and currently available commercial software for automated data capture and analysis, would take 7–10 days, at 7 h per day (not shown), whereas the overall CometChip Platform, utilizing the Celigo imaging system combined with the CAS, can capture and quantify upwards of 40,000 comets per plate in 30 min. Even without the advantage of the Celigo imaging system, only a maximum of 60 min is required to capture the images in the plate since all are in the same z-plane and there are no comet overlaps. With the CAS for analysis, this brings the time for capture and analysis to a maximum of 90 min, a major advance over the traditional slide-based comet assay. A key improvement over the original cometchip software is automated compatibility for use on any microscope or imaging platform. Together with enhanced hardware, these advances increase throughput by more than 100-fold, advancing the utility of the CometChip Platform for DNA damage analysis.

We thoroughly tested this next-generation platform for accuracy and reproducibility, quantifying >2 × 10^6^ comets during the course of this study. This analysis included the assessment of known DNA damaging agents. Using this approach, we verified that the effect of a number of common DNA damaging agents could be accurately measured using the CometChip Platform. We also determined limitations of the new system. In particular, the CAS had some limitations on the amount of DNA damage that could be measured in a cell. We determined that the upper limit of % tail DNA was consistently between 70–80%. Beyond this level of DNA damage, the comets became increasingly dim and the tails became so large and rounded that the software could not distinguish the tail and the remaining comet head. This has the unwanted effect of causing an artificial reduction in detecting the full range of DNA damage. Nevertheless, we maintain that this upper limit is not a failure in the software or CometChip Platform but inherent to the assay itself. Once the upper limit of approximately 80% is reached, the traditional comet assay itself becomes less and less quantitative, as has been universally observed in studies using the traditional comet assay. In this study, the upper limit was most clearly evident in the pre-screening result for compound F9 (tribromoacetaldehyde). At the highest concentration, treatment with tribromoacetaldehyde caused so much DNA damage that the CAS could not accurately quantitate the data due to failure to distinguish comets with % tail DNA beyond the 80% limit. This resulted in fewer than expected comets identified and large error bars. While it was possible to manually select and exclude incorrectly labeled comet images, we found that this also excluded comets with high DNA damage and again resulted in DNA damage values that were artificially low. In this study, exclusion of comets from the data set was limited to rare comets that clearly showed multiple side-by-side cell loading, affecting quantitation. As detailed in the “Assessment of CometChip loading” section, each cell type needs to be evaluated for the optimum cell concentration for loading and the optimum micropore size to avoid multiple cells loaded per well.

Relative to the traditional comet assay, a major advance represented by the CometChip Platform is the reduction of inter- and intra-assay variability. This has been a confounding factor in the traditional comet assay, limiting its use in population studies, particularly in collaborations involving multiple research groups. The original cometchip showed the promise of the microarray format for reducing experimental noise^23^, and this concept has been extended here. Building on our promising results with patient-derived samples, the CometChip can be equipped with a range of onboard controls, opening the door for large-scale screening of population cohorts using this method, thus providing new opportunities in human biomonitoring opportunities.

The majority of experiments conducted in this investigation were done under alkaline (pH < 13) conditions. This protocol was chosen as it allows for the processing of not only double-stranded DNA breaks (that can also be detected under neutral conditions) but also single-stranded DNA breaks (SSBs) and apurinic/apyrimidinic (AP) sites, both common forms of DNA damage and base excision repair intermediates^54^. The ability to detect SSBs and AP sites becomes particularly relevant when considering the repair capacity of Polβ-deficient cells^55^. We were interested in the DNA repair capacity of the methylating agent MMS because it is an S_*N*_2-class alkylating agent that requires the dRP lyase activity of Polβ for repair^31^. Hence, in the absence of Polβ activity, we would expect higher levels of SSBs and/or AP sites (repair intermediates) detectable by the CometChip assay. Indeed, Polβ-deficient mouse embryonic fibroblast (MEF) cells show elevated levels of DNA damage after MMS treatment and repair^55^. We replicated the same experiment herein and also found the Polβ-deficient HCT116 cells had a higher level of DNA damage 2 h post-exposure than wild-type cells; however, the difference was more modest than results previously reported^55^. Whether Polβ-deficient cells are also sensitive to H_2_O_2_ is debatable, with the weight of evidence suggesting that deficient cells either are not sensitive^32^ or only moderately sensitive^56,57^. When comparing the Cas9 expressing parental line to Polβ-deficient HCT116 cell lines, we also report limited difference in DNA damage induction and repair after H_2_O_2_ treatment. While not directly tested in this study, the observation that Cas9 expressing cells had a DNA damage level above baseline, particularly after MMS treatment, but to a lesser extent H_2_O_2_ exposure, highlights that caution should be used when using CRISPR-mediated knockout cells in DNA repair research, especially for cells that constitutively express the Cas9 nuclease.

There remains an unmet demand in the field of molecular biology and population science/epidemiology for a high-throughput technique that accurately and reproducibly measures DNA damage and repair. Such an assay would be ideally suited to be used as a complement to additional assays (Ames test, micronucleus, chromosomal aberration) when evaluating potential genotoxicants. Here, we utilized the CometChip Platform in a number of different applications to show that this assay can indeed fill many of these research requirements. We also conducted a proof-of-principle experiment to show that the CometChip could be used to screen a library of 74 compounds (potential DNA damaging agents) supplied by the NTP. With a short, 30 min exposure at a single dose of 100 μM, we found that 13 out of the 27 known genotoxicants in this library were direct DNA damaging agents, as revealed by a positive response in the CometChip screen (Table 1). These 27 genotoxic compounds act through a variety of modes of action (Tables 2 and 3), and not all are expected to induce primary (direct) DNA damage after an acute, low dose exposure, although all are classified as genotoxic based on data obtained in alternative assays, including assays for induction of mutations in bacteria (Ames), chromosomal aberrations in cultured mammalian cells, and micronucleus induction in rodents (Table S3). For example, published data for 4-(dimethylamino)azobenzene indicates it induces the formation of 8-hydroxyguanine (8-OH-Gua) in the livers of exposed mice^58^ and would therefore be classified as genotoxic since it induces base damage that leads to an increase in mutations. However, the base lesion 8-OH-Gua would not necessarily be detected in the comet assay without first treating the DNA with the OGG1 glycosylase (the FLARE-Comet assay)^59^. However, aborted or incomplete repair of the 8-OH-Gua lesion by the base excision repair pathway would be revealed as a single-strand DNA break. Also, 4-(dimethylamino)azobenzene requires metabolic activation (rat liver S9) for positive responses in NTP bacterial and mouse lymphoma TK5178Y^+/−^ cell mutagenicity assays (see Table 3). Previous analyses of cells treated with colchicine^60^ and diglycidyl resorcinol ether (DGRE)^40^ suggest the need for longer exposures and higher doses to detect DNA damage. Notably, however, DGRE was more cytotoxic to chicken DT40 cells knocked out for *Fanc-C*, a gene that is essential for the repair of DNA interstrand crosslinks, than to wildtype DT40 cells^40^. This observation, along with the fact that DGRE has two highly reactive epoxide groups, strongly indicates that DGRE damages DNA by a crosslinking mechanism and would be expected to reduce DNA migration in the comet assay. Indeed, we show in Fig. S4 that the % tail DNA for DGRE was markedly lower than the DMSO control. Colchicine, another of the genotoxicants that was negative in the CometChip experiment, is a spindle fiber poison, inducing aneuploidy; it is not a classic DNA damaging agent and has been shown to be negative in 4 out of 5 of the Tox21 DNA damage assays (Table S3).

Chromosomal damage induced by 17*beta*-estradiol has shown cell type specificity, and requires prolonged exposure and metabolism^46^; it is negative in the Ames assay and the rodent micronucleus assay^47^. In addition, black cohosh extract, although listed as a genotoxicant based on an *in vivo* micronucleus assay in rodents (Table S3), may act through either an aneugenic or clastogenic mechanism^61^, with the former not expected to be associated with DNA damage (and indeed, black cohosh is non-mutagenic in the Ames assay) (Table 3). Interestingly, glycidol was inactive in all 5 Tox21 DNA damage assays (Table S3), suggesting that the 100 µM top concentration may be a factor in the negative results obtained with this compound. This is consistent with reports showing the induction of DNA damage by glycidol requires a dose of 10 mM, a 100-fold increase in dose^62^.

Other agents listed in Table 3 also require higher doses and longer exposure times than were used in the acute, single dose pre-screen. The compounds azidothymidine and 6-thioguanine are nucleoside analogs that must be incorporated into DNA to reveal DNA damage. This requires higher doses and treatment times of 24 h or more *in vitro*^41,43^; *in vivo*, exposure times of 7 days^42^ are required to reveal evidence of DNA breaks. Ethyl methanesulfonate (EMS) and methyl methanesulfonate (MMS) are well documented alkylating agents but as reported elsewhere (and as shown herein), require longer exposure times and/or much higher concentrations (>2 mM for EMS) to show measurable DNA damage by the comet or CometChip assay (Table 3)^45^. An important caveat in the use of longer treatment times is the possibility of introducing secondary effects, including blocks to replication, transcriptional defects, or mitochondrial dysfunction. Such off-target effects might suggest the compounds are DNA damaging agents but this interpretation could be misleading. However, when we retested the 14 genotoxicants that were negative in the initial CometChip prescreen using a 24 h exposure duration, only three compounds (4-(dimethylamino)azobenzene, EMS, and 2-acetylaminofluorene), induced increases in DNA damage (Fig. S4). In addition, DGRE showed a strong reduction in DNA migration, suggesting that this agent is indeed a DNA crosslinker. Note however that a 24 h exposure was quite cytotoxic for many of the compounds, especially colchicine (>95% toxicity at 10 μM). Taken together, 92% (13/14) of the 27 known genotoxicants that were expected to increase DNA damage in the CometChip behaved as expected, and 100% (10/10) of the 27 known genotoxicants that were expected not to produce a response in the CometChip due to mode of action, a requirement for metabolic activation, or exposure conditions (concentration and duration of exposure) behaved as expected (Table 3).

Hence, we have demonstrated that a CometChip Platform pre-screen (single dose, acute treatment) may be used to identify DNA damaging agents, but not all genotoxicants will be detected using this method, based on mechanism of action. As recently described^63^, a thorough analysis of genotoxicants requires evaluation by a combination of tests (Comet/CometChip, Ames test, micronucleus, chromosomal aberration), each providing different mechanistic evaluations and revealing genotoxicity profiles based on different modes of action. We suggest that compounds that are negative in a CometChip Platform pre-screen should be subjected to more extensive testing that includes multiple doses and treatment times, in concert with additional assays (Ames test, micronucleus, chromosomal aberration). As with all existing genotoxicity assays, no single assay is capable of detecting all genotoxic agents. The CometChip technology, however, provides a means of quickly identifying DNA damaging agents among a large library of uncharacterized compounds, thus playing a valuable role in reducing the number of environmental agents and industrial compounds for which no data currently exists.

The CometChip Platform described herein significantly increases the quantity of samples that can reasonably be analyzed and enables researchers to ask more complex questions since the number of samples (cell or chemical samples) can be extensive and analysis time is markedly reduced. These features will allow detailed analysis of environmental genotoxicants as well as chemotherapeutic agents in future studies that may help glean information on exposure and mechanisms of DNA damage and repair as well as clinically relevant data about new and existing DNA damaging compounds. In addition, basal and induced DNA damage levels can be ascertained in support of population studies.

## Methods

### Compounds

H_2_O_2_, MMS, MNNG and etoposide were obtained from Sigma-Aldrich (St. Louis, MO). Compounds from the NTP were provided by the NTP Chemistry Group as frozen, identity-masked 20 mM stock solutions in DMSO (known genotoxicants shown in Table S3). Compounds were stored at −20 °C until use. Additional DMSO from the NTP supply was provided for making dilutions of the chemical stock solutions. Detailed chemical analyses have been conducted to confirm identity and purity of the compounds in the Tox21 10,000 compound library^35,64^; results of the analyses for the NTP compounds used in this study are included in Tables 1 and 2.

### Cell culture

TK6 (ATCC® CRL-8015™) cells are p53 proficient lymphoblasts derived from a human male. Jurkat, Clone E6–1 (ATCC® TIB-152™), cells are lymphoblast cells derived from a human male with acute T cell leukemia, they have a mutation in the *TP53* C-terminal domain responsible for transactivation, resulting in a defective p53-signaling pathway^65^. Both cell lines were purchased from the American Type Culture Collection (ATCC; Manassas, VA) and were cultured as per ATCC instructions in RPMI-1640 media with 10% fetal bovine serum, supplemented with 1% penicillin-streptomycin in 5% CO_2_ at 37 °C.

HCT116 cells are a human colorectal carcinoma cell line deficient in mismatch repair (MMR) but are p53 proficient. The HCT116 cell line was purchased from ATCC (ATCC^®^ CCL-247™ ATCC) and was cultured as per ATCC guidelines in McCoy’s 5 A media with 10% fetal bovine serum and supplemented with 1% penicillin-streptomycin in 5% CO2 at 37 °C. HCT116/p53-KO cells were a generous gift from B. Vogelstein (J. Hopkins Univ). A dual plasmid (Addgene #52963, #52962) lentiviral vector^66^ was used to facilitate CRISPR-Cas9 mediated mutation of the POLB gene. Two alternate guideRNA (gRNA) pairs were used: Polβ 1.3–5′GAGCAAACGGAAGGCGCCGC predicted to cut at genomic location chr8:42338643 in exon 1 of the gene, Polβ 1.5–5′CGCCGCAGGAGACT CTCAAC predicted to cut at chr8:42338657 in exon 1 of *POLβ*. Control cell lines expressing Cas9 were created using the #52962 plasmid without gRNA insert. Lentiviral transduction protocol was as previously described^28^. Cells were maintained in media containing the puromycin selection agent (2.5 μg/ml) for 12 days before immunoblot protein validation of the knockout was performed.

The MDA-MB-231 human breast adenocarcinoma cell line was used to measure the efficiency of cell loading into the CometChip. The cells were stably transduced with lentivirus expressing either EGFP or RFP and grown under puromycin (5 μg/ml) selection for 7 days. Cells were cultured in Leibovitz L-15 media supplemented with 10% FBS, 1% penicillin-streptomycin in 5% CO_2_ at 37 °C. The parental MDA-MB-231 cells were kindly provided by Dr. Julie Eiseman (University of Pittsburgh).

### CometChip Platform and Supplies

The CometChip Platform is now available from Trevigen, a division of Bio-Techne (Minneapolis, MN) and includes the disposable 30 µM CometChips, the CometChip 96-well magnetically sealed cassettes (formers), the CometChip Electrophoresis System (CES) and the Comet Analysis Software (CAS).

### General CometChip protocol

Cell size was measured using the Countess® II automated cell counter (ThermoFisher Scientific; Waltman, MA) to ensure selection of cells appropriately sized for the CometChip microwells. The 30-micron sized CometChip (Trevigen, Gaithersburg, MD) was used in all experiments with the exception of the comet loading experiments. Each well in the 96-well CometChip contains approximately 500 microwells. Cells were loaded into the CometChip apparatus at a concentration of 50 K cells per well. Cells were gravity loaded into the microwells for 30 min with the CometChip and former placed in the cell culture incubator (37 °C, 5% CO_2_). Treatments were conducted in the CometChip with incubation taking place in a cell culture incubator. Compounds of interest or vehicle controls were diluted in full media and applied immediately after the cells were loaded into the CometChip. After treatment (e.g., H_2_O_2,_ MMS, MNNG or etoposide), the chip was washed multiple times with PBS and sealed with low melting point agarose (LMPA) (Topvision; ThermoFisher Scientific) (7 ml; 0.8% LMPA/PBS). The CometChip was then submerged in lysis solution with detergent (Trevigen) for 40 min at 4 °C. The CometChip was run under alkaline (pH > 13) conditions (200 mM NaOH, 1 mM EDTA, 0.1% Triton X-100). Electrophoresis was conducted at 22 V for 50 min at 4 °C. For the 24 h screening experiments, electrophoresis was conducted at 28 V for 25 min with reduced buffer volume. After electrophoresis, the CometChip was re-equilibrated to neutral pH using Tris buffer (0.4 M Tris·Cl, pH 7.4). Subsequently, the DNA was stained with 1 × SYBR Gold (ThermoFisher Scientific) diluted in Tris buffer (20 mM Tris·Cl, pH 7.4) for 30 min and de-stained for 1 h in Tris buffer (20 mM Tris·Cl, pH 7.4).

### Automated image acquisition

Image acquisition was conducted on the Celigo S imaging cytometer (Nexcelom Bioscience, Lawrence, MA) at a resolution of 1 micron/pixel with whole plate imaging to avoid imaging variability. Image analysis was conducted using the dedicated CAS (see below) with the box size set to 220 × 180 pixels which represented a box size that would capture comets from heavily damaged cells without box overlap. Data acquired were exported to Excel (Microsoft) and subsequently to Prism 7 (GraphPad Prism) for statistical analysis.

### Comet loading controls

MBA-MD-231 cells were transduced with lentivirus expressing either green (EGFP) or red (RFP) fluorescent protein, as we have described previously^27^. Cells where then loaded into the CometChip with multiple well sizes (20, 30 and 40 μm) at multiple concentrations ranging from 10^2^ to 10^5^ cells/ml. The plate was then washed to remove cells that had not settled into a well and imaged on an EVOS XL imaging system (ThermoFisher Scientific). Images were taken using the green (470EX/510EM) and red (531EX/593EM) channels and also merged to detect wells that contained multiple cells.

### CometChip inter- and intra-assay variability

Jurkat cells were treated with etoposide (5 μM) for 1 h and the CometChip processed as per the above general protocol. Percent tail DNA was measured in each well across rows E and F and each well across columns 6 and 7. Results from plate rows E and F were combined to give a single value, as were the results from columns 6 and 7 (Fig. 3A). Mean values were graphed showing 95% confidence interval (CI). Inter-assay variability was measured as a component of the compound screening assay described below. Each compound screened also had an internal positive DNA damage control run on the same CometChip. This series of experiments was run over a period of three months by three different investigators resulting in 17 separate CometChip-derived datasets for each cell line.

### Assessment of known DNA damaging agents

Jurkat and TK6 cells were exposed to four known DNA damaging agents: etoposide, H_2_O_2_, MNNG and MMS, at concentrations ranging from 0.1–100 μM for 1 h at 37 °C. Cells were then analyzed using the general CometChip protocol. Assays were conducted in duplicate. DMSO was used as the vehicle control in all experiments at a concentration correlating with the highest % of DMSO in any sample. In all experiments, DMSO was kept at or below 0.1% total sample volume.

### Endogenous DNA damage and the measurement of DNA repair kinetics

CRISPR-mediated knockout of DNA Polymerase β (method detailed above) was confirmed by immunoblot. In brief, whole cell protein extracts were isolated from HCT116 cells and separated on a 4–12% SDS-PAGE gel (Novex, Invitrogen, ThermoFisher Scientific). Separated proteins were transferred to a PVDF solid membrane support using the BioRad turbo blotting system (BioRad, Hercules, CA). Membranes were then blocked in 4% milk before overnight exposure to primary antibodies: anti-DNA polymerase β (1:500) (Clone 61, MA5–12086, ThermoFisher Scientific), anti-CRISPR-Cas9 (1:1000) (7A9–3A3, NBP2–36440, Novus; Littleton, CA) and anti-α-tubulin (1:1000) (CP06, Calbiochem; Burlington, MA). Targeted proteins were visualized using chemiluminescence, detected using a BioRad Chemidoc System. For repair studies, cells were exposed to MMS (1 mM) or H_2_O_2_ (50 μM) for 30 min, wells were washed twice with media without FBS before the addition of original (depleted) growth media. All experiments were conducted at 37 °C with 5% CO_2_ in a humidified incubator.

### Patient derived lymphocytes

A healthy, non-smoking male volunteer was consented and blood was obtained via the MCI Biobank. These samples and all methods to obtain and isolate these patient derived lymphocytes were obtained in accordance with the relevant guidelines and regulations of the University of South Alabama (USA) Institutional Review Board (IRB). All experimental protocols for the isolation and preparation of the patient derived lymphocytes were approved by the USA/IRB committee – as defined in the USA/MCI protocol # 03–092. As required, informed consent was obtained from the subject. Once obtained, the blood was transferred into BD vacutainer CPT mononuclear preparation tubes (BD) containing the anti-coagulant sodium heparin and blood separation media composed of a thixotropic gel and FICOLL™ Hypaque™ solution. Samples were centrifuged at RT at 1500 relative centrifugal force (rcf) for 15 min using a swinging bucket rotor (5810 R, Eppendorf, Hauppauge, NY). After centrifugation, the lymphocytes and mononuclear cells were visible as a white layer that was collected using a Pasteur pipette. Average cell size and viability (trypan blue exclusion) were measured using the Countess II automated cell counter (ThermoFisher Scientific). An average of four readings were taken for each sample. Samples were loaded into the CometChip and exposed to H_2_O_2_ (200 μM) or etoposide (5 μM) for 30 min.

### NTP Compound Screen

The compound screen was conducted initially using Jurkat cells loaded into the CometChip. After loading, the cells were treated with the target compound (100 μM single concentration, 30 min) in RPMI medium without phenol indictor, antibiotics, or FBS. Subsequent tests on subsets of the NTP compounds (1 h exposure over a concentration range of 1–25 μM in both the Jurkat and TK6 cells) were conducted in a similar fashion. Treatment was conducted at 37 °C and 5% CO_2_. For the 24 h exposure at a dose of 10 μM in TK6 cells, cells were treated in 10 ml flasks at 37 °C and 5% CO_2_ and cells were then loaded into the CometChip. Following treatment, the CometChip was sealed (0.7% LMA/PBS) and subject to electrophoresis. The CometChip was imaged using the Celigo S imaging platform as described above. The CometChip data were then analyzed using the Trevigen CAS, which measures a range of commonly used parameters including % tail DNA, tail length, area, and intensity of tail. For all data, % tail DNA was used to measure the amount of DNA damage. Significance was measured using a one-tailed students t-test when comparing all compounds to the control values. In all experiments, a positive control of Jurkat/TK6 cells exposed to etoposide (5 μM) was included to allow for more accurate comparison across CometChips. In all experiments, DMSO was kept at or below 0.1% total sample volume.

### Statistical analysis

Statistical analysis was conducted using Prism 7 software (GraphPad, La Jolla, CA). Percent tail DNA was graphed as mean ± 95% confidence interval (CI). Significance was measured using unpaired two-tailed students t-test when comparing only two groups (Figs 3B, 6C and 7C). When comparing three or more groups, we measured significance using one-way ANOVA (Fig. 5B). For the data presented in Fig. 7A and Tables 1 and 2, significance was measured using a one-tailed Student’s t-test when comparing all compounds to the control values. Throughout the manuscript, duplicate testing refers to two biological replicates.

### Data and materials availability

All data and materials are available upon request.

## Electronic supplementary material

Supplementary Materials

Supplementary Dataset 1

Supplementary Dataset 2

Supplementary Dataset 3

Supplementary Dataset 4
