# Supplementary material for: Next generation high throughput DNA damage detection platform for genotoxic compound screening
Source: Sci Rep. 2018 Feb 9;8:2771. doi: 10.1038/s41598-018-20995-w (PMC5807538; doi:10.1038/s41598-018-20995-w)
Supplement: Supplementary file 1 — Supplementary Materials [file 41598_2018_20995_MOESM1_ESM.pdf]

Next generation high throughput DNA damage detection platform for genotoxic compound screening

SUPPLEMENTARY MATERIALS

Short title: Next generation DNA damage detection platform

Authors:

Peter Sykora[1], Kristine L. Witt[2], Pooja Revanna[1], Stephanie L. Smith-Roe[2], Jonathan Dismukes[1], Donald G. Lloyd[3], Bevin P. Engelward[4] and Robert W. Sobol[1*]

Affiliations:

[1]Department of Oncologic Sciences, Mitchell Cancer Institute, University of South Alabama, 1660 Springhill Avenue, Mobile, AL 36604

[2]Division of the National Toxicology Program, National Institute of Environmental Health Sciences, Research Triangle Park, NC 27709

[3]DGL-Imaging, Millersville, MD, 21108

[4]Department of Biological Engineering, MIT, Cambridge, MA 02139

***Address correspondence to**:
Robert W. Sobol, PhD
Mitchell Cancer Institute, University of South Alabama
1660 Springhill Avenue, Mobile, AL 36604
Tel: (251) 445-9846
Lab: (251) 445-9893
E-mail: rwsobol@health.southalabama.edu

Image acquisition and Comet Analysis Software (CAS) – detailed description.

The CAS analyzes comet prep images by the systematic application of a carefully configured series of mathematical image processing techniques. These processing techniques are designed to maximize processing efficiency and at the same time assure analytic accuracy and reliability.

A digitally collected comet image is essentially comprised of a two-dimensional array of numbers organized into an indexed number of rows and columns. The numeric values corresponding to the intersecting positions of the rows and columns are commonly referred to as pixels or picture elements. Each pixel provides a one-to-one mapping to the fluorescent light intensity displayed and microscopically measured at a corresponding spatial location in a real scene of interest on a comet slide or chip. Image processing techniques refer to mathematical operations or algorithms applied to such a numeric pixel array, to derive particular analytic results.

The image processing techniques employed by the CAS seek first to partition or segment fluorescent objects of potential interest from the image background using a scanning search box coupled with an adaptive thresholding routine (**Fig. 2B**). The search box size is set such that it can encompass individual comets. The default height of the search box is typically set at 2.3 times the diameter (D) of a typical comet head, as measured *a priori* within a negative control prep. The default box width is set at 4 times D. The intensity threshold used by the search routine is set dynamically to a value that is just nominally above the intensity displayed by the image background. The background intensity is determined from the modal (i.e. most frequent) value in an intensity histogram drawn locally from the surrounding region in which the search box is scanning. The background represents the largest area feature in the image and thus yields the modal value in the histogram. Regionalizing the histogram to an area around the search box position allows the search routines to adjust for variations in background intensity, such as changes in non-specific background fluorescence across the image.

The CAS search routine is designed to locate any contiguous fluorescent region or cluster of pixels in the image that is above the established intensity threshold, and fits in the scanned search box. A located object may simply correspond to a bit of fluorescent debris in the scene or it may correspond to an actual cell (comet) requiring further detailed analytic attention to obtain related measures of DNA damage. In general, hundreds or thousands of candidate objects will be found within a typical image. It is, therefore, important from both a data accuracy perspective and from a processing efficiency perspective that the CAS incorporates a methodology to quickly eliminate non-target objects, such as debris, from consideration and to reliably identify objects (cells) that can be appropriately analyzed for DNA damage.

To accomplish this, the CAS systematically derives a series of quantitative measures, which characterize found objects in terms of various dimensions of a classifier model. Quantitative limits related to each analytic dimension allow the finding routines to classify a candidate object as either consistent with the properties of a comet or inconsistent, and therefore a non-target object to be rejected from further consideration. While the total process is complex, the underlying classification is conceptually simple. The CAS simply asks and answers questions such as:

- Is this object that was found too small to be a comet?
- Is it too big?

· Is the long axis of the object oriented in a direction consistent with the direction of electrophoresis?
· Is the object's shape consistent with the expected shape of a comet?
· Is the object essentially symmetric about a horizontal line drawn through its center?

By asking and answering such questions, the CAS systematically sieves through the objects found on each image and identifies them as non-target objects to be rejected from further analysis or as analyzable cells (comets) requiring further attention.

To illustrate some of the measures and types of criteria used by the CAS to answer such questions and perform its classification of objects, consider an arbitrary object found in an image.

This object will have some vertical extent or height, as indicated by the number of rows in the image array that it spans. Similarly, a measure of its horizontal width is indicated by the number of columns in the image array that the object spans. The classification routines compare these object size measures against various length criteria to determine if the subject object is too small or too large to be an analyzable comet. For example, if either the height or width of the subject object is less than a default minimum of 0.3 times D, the classification routine will reject the object as too small. If the height is greater than 0.8 times the search box height or if the object width is greater than 0.9 times the search box width, then the object is rejected as too big. Objects that escape rejection on the basis of size are then evaluated on the basis of aspect or orientation. This is accomplished by dividing the object's width by its height to yield an aspect ratio. Objects that are elongated in the vertical direction will exhibit aspect ratios that are less than one. Objects that are round or oval will exhibit aspect ratios that are close to one. And, objects that are elongated in the horizontal direction will exhibit aspect ratios that are greater than one. Images analyzed by the CAS are to be collected such that comet heads are oriented to the left with tails (if any) streaming horizontally to the right. Consequently, the classification routines are configured to pass objects that have aspect ratios near or greater than one, and reject objects whose aspect ratios are significantly less than one. A default value of 0.7 is typically used to define the dividing line between object rejection and acceptance.

Finally, the classification routines perform a more refined analysis of the shapes of the surviving objects. Analyzable comets will exhibit reasonable symmetry about a line drawn horizontally through their center. Significant deviations from such symmetry, however, can signal problems that would adversely impact the derivation of reliable quantitative measures of DNA damage. Such problems may include incomplete DNA staining, overlap of multiple cells, contamination of comet heads or tails with fluorescent debris, and misalignment of images such that comet tails are streaming in a diagonal direction. In order to quantitatively evaluate the degree of symmetry exhibited by candidate objects, the system utilizes a spatial adaptation of a familiar measure used in the field of statistics, Pearson's 2nd coefficient of skewness (P_{2s}), which is computed as:

$$P_{2s} = 3\ (\text{Mean} - \text{Median})\ /\ \text{Standard Deviation}$$

This statistic measures the relative symmetry or asymmetry of a distribution and has a range from -3 to 3. For reasonably symmetric distributions, it takes on values at or close to zero. The further from zero the measure is, the greater the skew or asymmetry of the subject distribution being evaluated. A positive or negative sign signals the direction of the distributions skew. The

CAS applies this measure to strategically positioned cross-sections of the object under consideration (i.e. the columnar data) and to an aggregate distribution (obtained by summing the pixel values of the object horizontally along the rows), to probe for evidence of significant asymmetry. By default, objects that yield skewness measures that deviate from zero by more than 0.75 are rejected.

The surviving objects are judged to be consistent with the characteristics of comets and are then passed on to additional software routines, which analyze and quantify the associated distribution of fluorescent intensities and derive various measures of DNA damage (**Fig. 2C**). These additional routines first fit an interpolated surface to the background intensities surrounding a comet to model the local pattern of non-specific background fluorescence. They then subtract this fitted surface from the comet, to leave a corrected pixel intensity distribution that solely reflects the comet's DNA distribution. Strategic profiles and projections of the corrected intensity distribution are then generated and analyzed to identify the most likely position of the dividing line between the head and tail region (if any) of the comet (seen as a red line, **Fig. 2C**), as well as the location of the terminal end of the tail (green line, **Fig. 2C**). These regional markers are then used to guide the straightforward computation of various measures of DNA damage, such as tail length, percent DNA in the tail (% tail DNA) and tail moment from the background corrected intensity distribution.

Protocol Files are used to store the various parameters required by the CAS to configure its search and analysis routines. The parameters stored in a Protocol file can be divided into two basic categories: parameters which are scale dependent, and parameters which are scale invariant.

The scale dependent parameters are sensitive to factors such as the electrophoresis time and voltage used, the objective magnification of the microscope, and the digital camera's sensor array type, size and resolution. For this reason, the CAS is equipped with a utility for making adjustments to existing Protocol Files and to define and save new files. These new Protocol Files can then be called up, when appropriate, to configure the search and analysis routines of the CAS. Examples of scale dependent parameters include the parameters used to define the search box size that is used when looking for comets in images. They also include various size criteria used to define if objects are too small or too large to be comets so that they can be quickly eliminated from consideration. Proper setting of these parameters depends on the apparent scale of comets as seen by the user's camera at a chosen objective magnification. Different cameras may see comets scaled differently, at the same microscope objective magnification, as a result of differences in the pixel count and array size of the camera sensor. The easiest way to adjust for such camera differences is to measure a typical (average sized) undamaged comet head in an image captured with the subject camera at the chosen magnification. The CAS can then automatically compute appropriate parameter values based on this measurement. The CAS, therefore, includes a utility that will walk a user through the head measurement process and calculate appropriate values for the various scale dependent parameters.

The scale invariant parameters are very stable and should only need to be adjusted in rare circumstances. The default values for these parameters have been optimized through extensive testing by Drs. George and Woodgate (Trevigen). If by accident any of these parameters were to get changed, there is a button that the user can click to reset the default values.

A number of pre-defined Protocol Files are supplied with the CAS and cover a range of comet system configurations. However, as indicated above, the user has the ability to fine-tune

or adjust any of the various parameters stored in a Protocol File, so as to optimize CAS performance for their particular comet system set-up.

Below is a summary of the key parameters stored in a Protocol File and specifies their permissible adjustment range and default values:

RANGE AND DEFAULT VALUES FOR PROTOCOL PARAMETERS

- CometBoxHeight
  - Range: Depends on image scale (2 – 3 times typical head diameter)
  - Default: 2.3 times typical head diameter
- CometBoxWidth
  - Range: Depends on image scale (3 – 5 times typical head diameter)
  - Default: 4 times typical head diameter
- MaximumCellColumns
  - Range: Depends on image scale (0.9 – 1.0 times CometBoxWidth)
  - Default: 0.9 times CometBoxWidth
- MaximumCellRows
  - Range: Depends on image scale (0.8 – 1.0 times CometBoxHeight)
  - Default: 0.8 times CometBoxHeight
- MinColsToRowsRatio
  - Range: 0.5 – 1.0
  - Default: 0.7
- MinimumCellColumns
  - Range: Depends on image scale (0.1 – 0.4 times typical head diameter)
  - Default: 0.3 times typical head diameter
- MinimumCellRows
  - Range: Depends on image scale (0.1 – 0.4 times typical head diameter)
  - Default: 0.3 times typical head diameter
- PercentCutoff
  - Range: 0.5 - 5
  - Default: 4
- PotentialCellMaxCutoff
  - Range: 245 - 255.
  - Default: 255
- PotentialCellMinCutoff
  - Range: 5 – 20
  - Default: 8
- SymmetryPercentTolerance
  - Range: 10 -35
  - Default: 25
- SymmetryStdDeviation
  - Range: 1- 4
  - Default: 2
- TailAsymmeticPercent
  - Range 20 -50
  - Default: 40

Supplementary Figures

Fig. S1A: Representative image of comets taken of HCT116 cells before (left) and after (right) etoposide treatment. Comet tail increases proportional to DNA damage (in support of **Fig. 1**).

Fig. S1B: To validate the CAS, mock comets were created with known parameters (in support of Fig. 2).

Fig. S1C: Analysis of artificially created comet data to establish the precision of the CAS algorithm. All three endpoints show strong relationship between calculated and CAS derived points (in support of **Fig. 2**).

Fig. S1D: Alkaline comet analysis of cells treated with etoposide (Alkaline Control Cells, Trevigen). Direct comparison of comets from the CometChip and the slide-based comet system using the Loats Automated Comet Assay Scoring System. Plots show comparison of %DNA in tail (top), Tail moment (middle) and comet length (bottom). Strong Correlation (~1) between CometChip and slide-based comet for damage parameters.

Fig. S1E, S1F: CometChip loading validation: **(E)** RFP and GFP expressing cells (20 μm mean diameter) were loaded into 20, 30 or 40 μm wells. Multiple cell loading into a single well increased proportionally with well size. CometChip loading: GFP and RFP cells were loaded into

the CometChip with varied well size (20,30,40 μm) **(F)** 100 wells that were filled with a GFP containing cell were checked to see if they also had an RFP containing cell. The proportion of wells that had more than one cell increased with well size. n=3. Error bars are mean ± SD. (in support of **Fig. 3**).

Fig. S1G, S1H: CometChip results from HCT116 cells that have been treated with etoposide for 1 hour. **(G)** Linear regression from treated WT and P53 mutant cells show that the CometChip assay can precisely measure DNA damage in the two cell lines. **(H)** CometChip can differentiate between concentrations of etoposide in HCT116 cells with a high degree of statistical certainty. ***= $p<0.001$, Error bars represent mean ± 95% CI of duplicate assays. (in support of **Fig. 3**).

Fig. S2: Full prescreen data. Jurkat cells were exposed to each compound at a concentration of 100 μM for 1 hr. Alkaline comet was conducted immediately after exposure. Control (NT) cells were vehicle treated. Also see **Fig. 7**. Error bars represent mean ± 95% CI. (in support of **Fig. 7**).

Fig. S3: Full screen data from the compounds identified as potentially genotoxic from the prescreen data (**Fig. 7**). Full screen was undertaken using Jurkat (circle) and TK6 (square) cells. Red symbols indicate the positive control (etoposide at 5μM) for each cell line. (in support of **Fig. 7**).

Fig. S4: TK6 cells were exposed to the non-DNA damaging agents listed in **Table 3** for 24 hours (10 μM). Assay was modified (electromagnetic field strengthened) to detect extremely small amounts of DNA damage. Three chemicals [2-acetylaminofluoride, $p<0.001$= ***; ethyl methanesulfonate, $p<0.01$= ** and 4-(dimethylamino)azobenzene, $p<0.0001$ =****] showed small but statistically significant increases in DNA damage. Di-glycidyl resorcinol ether (DGRE) showed a marked reduction in DNA damage, a response consistent with a crosslinking mode of action. Significance was measured using a one-tailed students t-test, comparing each compound to the DMSO control value. Etoposide was used as a positive control. n=2 biological replicates, n=4 technical replicates.

Supplementary Tables (Datasets)

Table S1: Data set of Intra-assay variability.

Table S2: Data set of Inter-assay variability.

Table S3: Data set - genotoxicity profiles of the known genotoxicants in the NTP compound library: responses in Tox21 DNA damage assays and in standard genotoxicity assays.

Table S4: Data set of One and Two-Tailed Students t-test for pre-screen results.

Supplementary Figures – Sykora et al.

Figure S1A

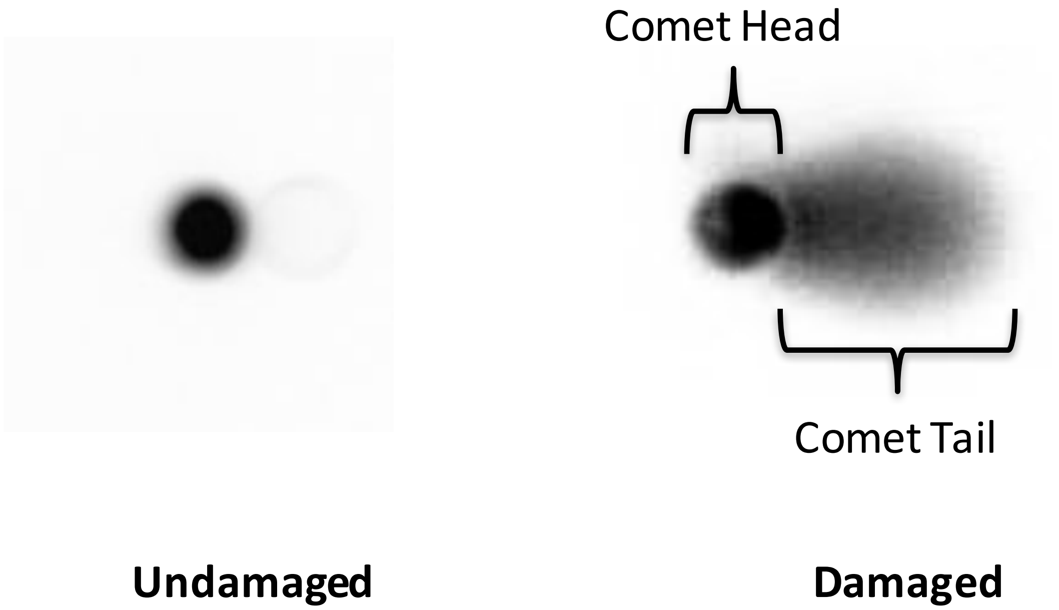

Supplementary Figure S1A to support Figure 1: Representative image of comets taken of HCT116 cells before (left) and after (right) etoposide treatment. Comet tail increases proportional to DNA damage.

Figure S1B

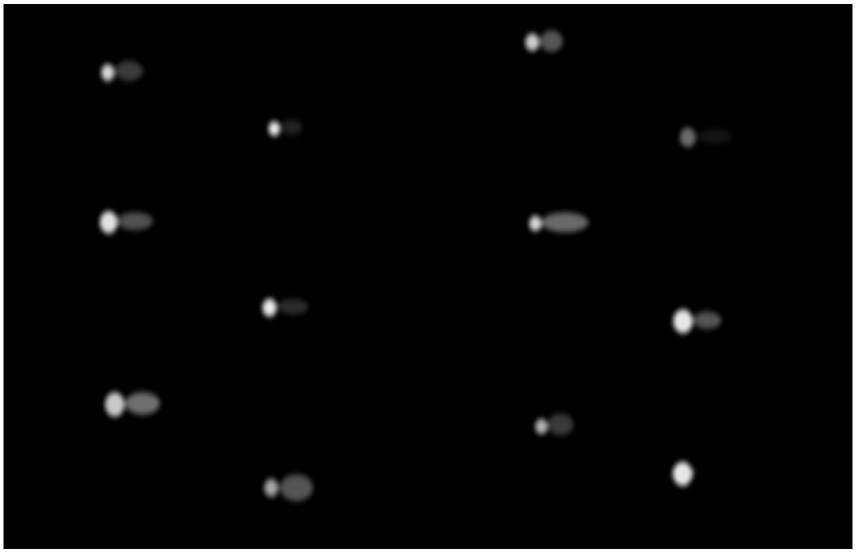

Supplementary Figure S1B to support Figure 2: To validate the CAS, mock comets were created with known parameters.

Supplementary Figures – Sykora et al.

Figure S1C

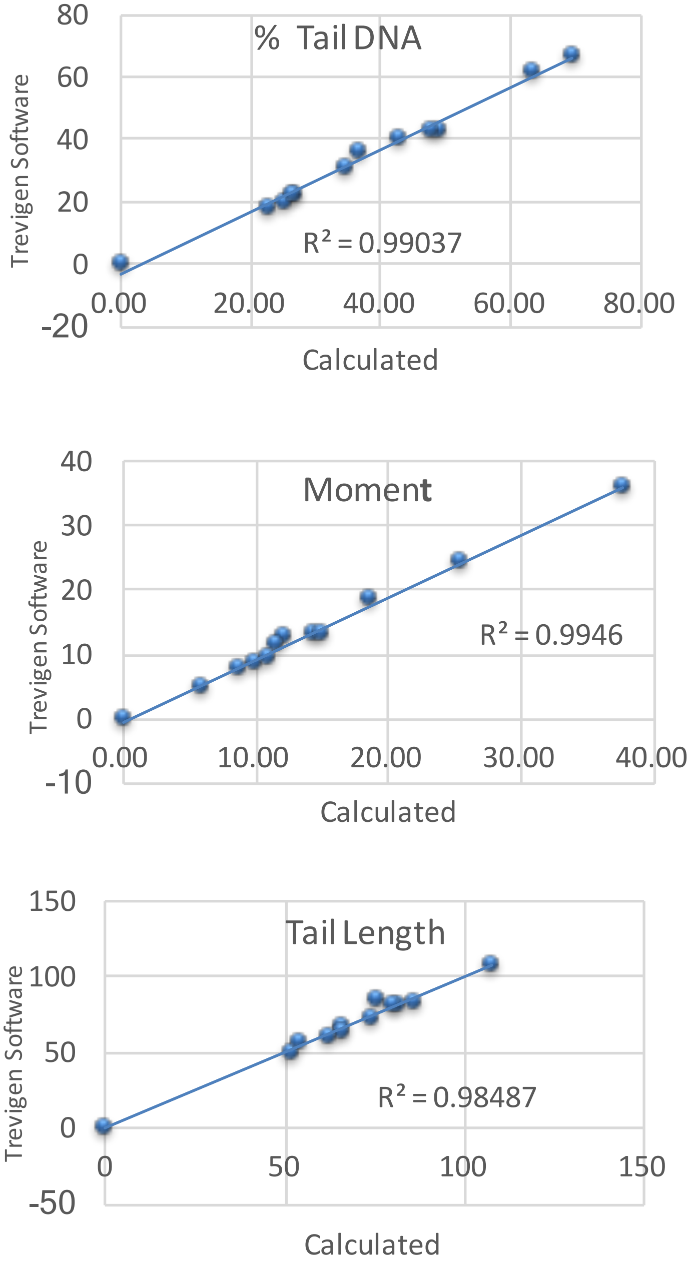

Supplementary Figure S1C to support Figure 2: Analysis of artificially created comet data to establish the precision of the CAS algorithm. All three endpoints show strong relationship between calculated and CAS derived points.

Supplementary Figure S1D: Alkaline comet analysis of cells treated with etoposide (Alkaline Control Cells, Trevigen). Direct comparison of comets from the CometChip and the slide-based comet system using the Loats Automated Comet Assay Scoring System. Plots show comparison of %DNA in tail (top), Tail moment (middle) and comet length (bottom). Strong Correlation (~1) between CometChip and slide-based comet for damage parameters.

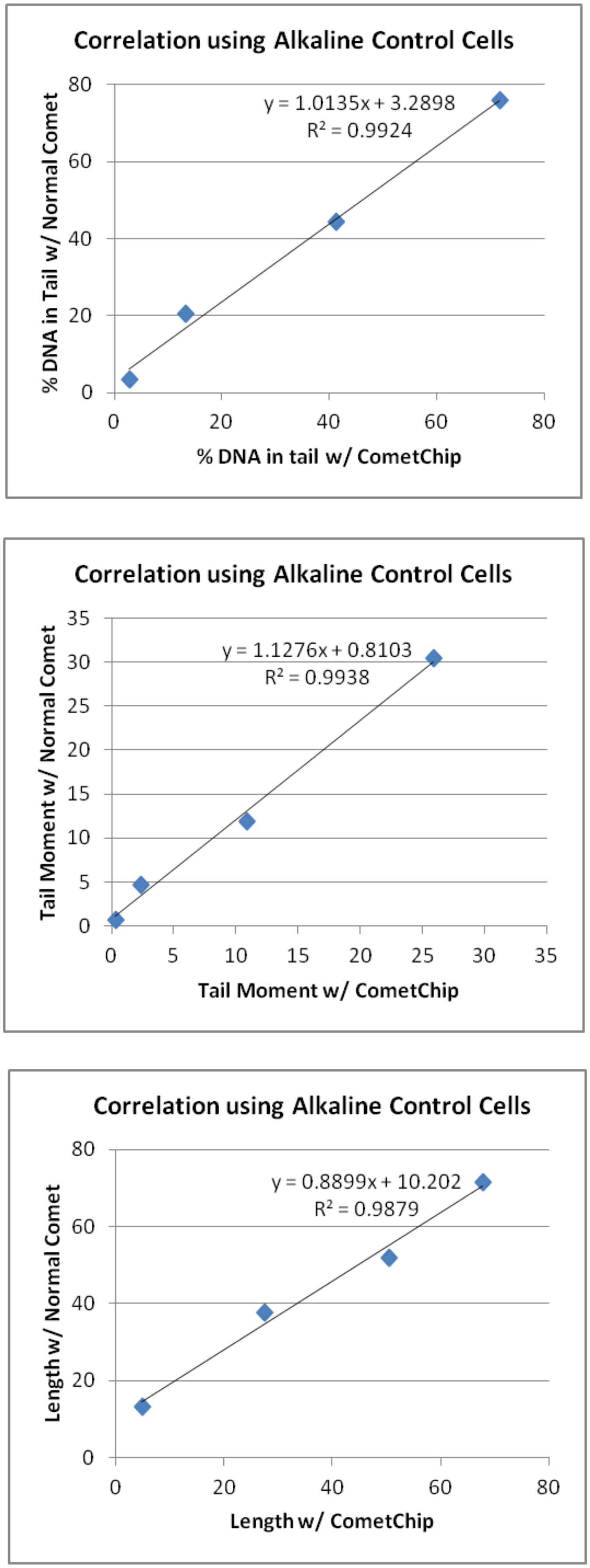

Supplementary Figures – Sykora et al.

Figure S1E

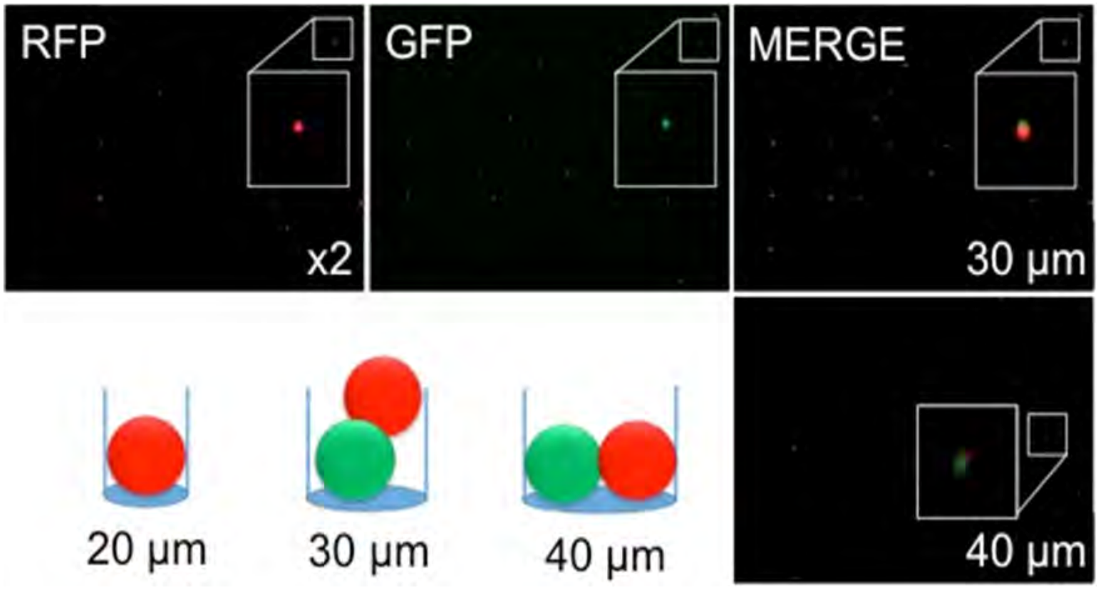

Figure S1F

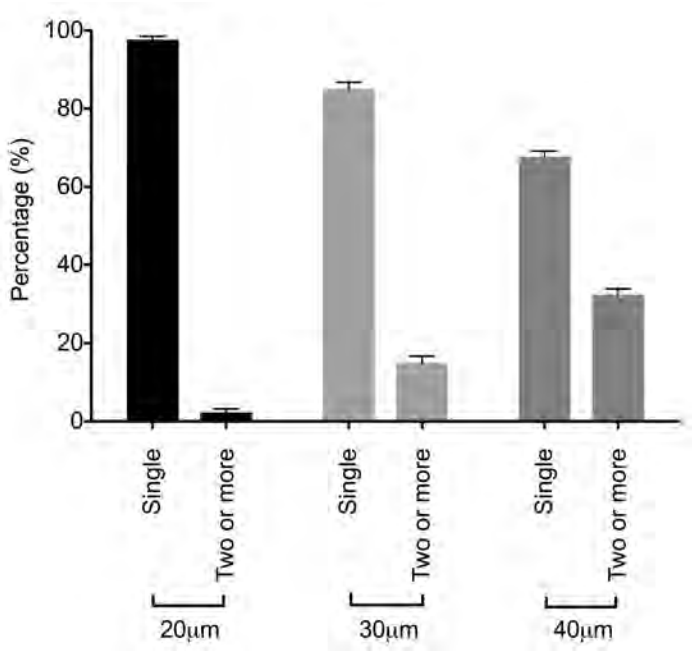

Supplementary Figure S1E, S1F to support Figure 3: CometChip® loading validation: **(D)** RFP and GFP expressing cells (20 µm mean diameter) were loaded into 20, 30 or 40 µm wells. Multiple cell loading into a single well increased proportionally with well size. CometChip loading. GFP and RFP cells were loaded into the CometChip with varied well size (20,30,40 µm) **(E)** 100 wells that were filled with a GFP containing cell were checked to see if they also had an RFP containing cell. The proportion of wells that had more than one cell increased with well size. n=3. Error bars are mean ± SD.

Supplementary Figures – Sykora et al.

Figure S1G

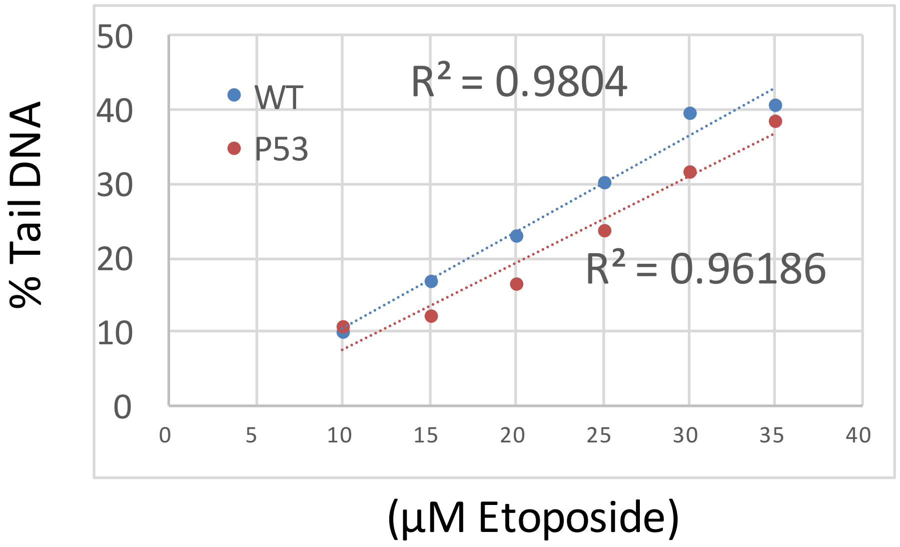

Figure S1H

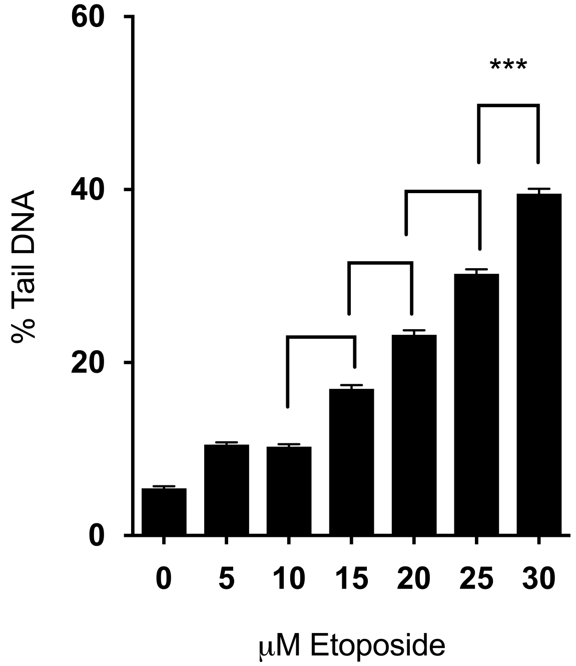

Supplementary Figure S1G and S1H to support Figure 3: CometChip results from HCT116 cells that have been treated with etoposide for 1 hour. **(F)** Linear regression from treated WT and p53 mutant cells show that the CometChip assay can precisely measure DNA damage in the two cell lines. **(G)** CometChip can differentiate between concentrations of etoposide with a high degree of statistical certainty. ***= $p<0.001$, Error bars represent mean ± 95% CI of duplicate assays.

Supplementary Figures – Sykora et al.

Figure S2A

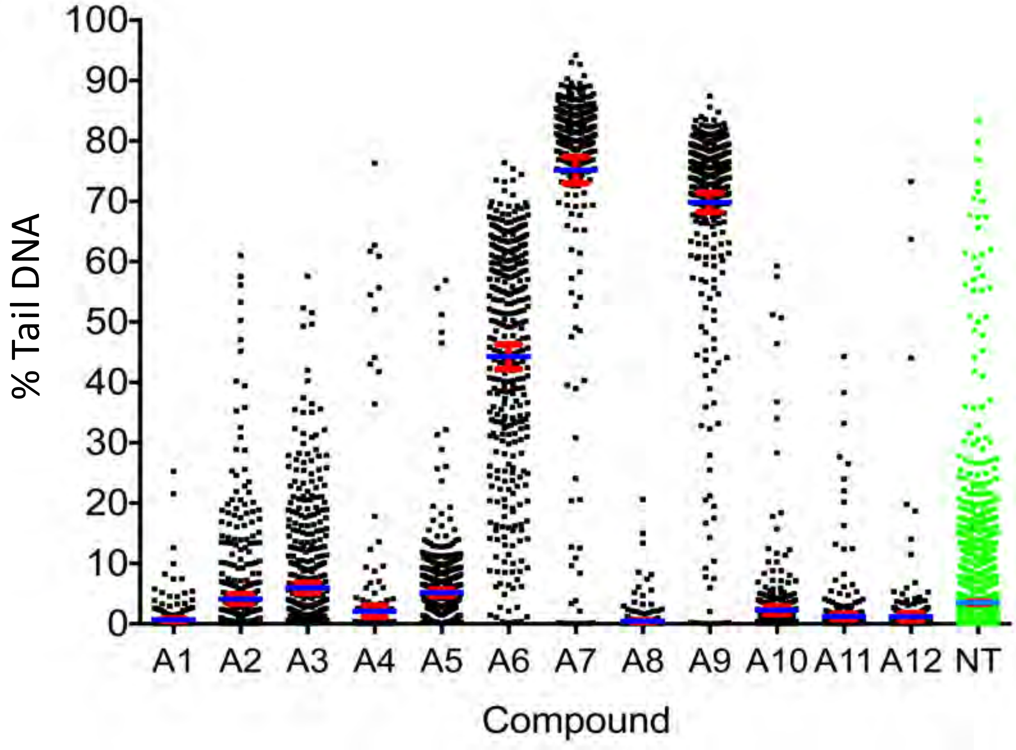

Figure S2B

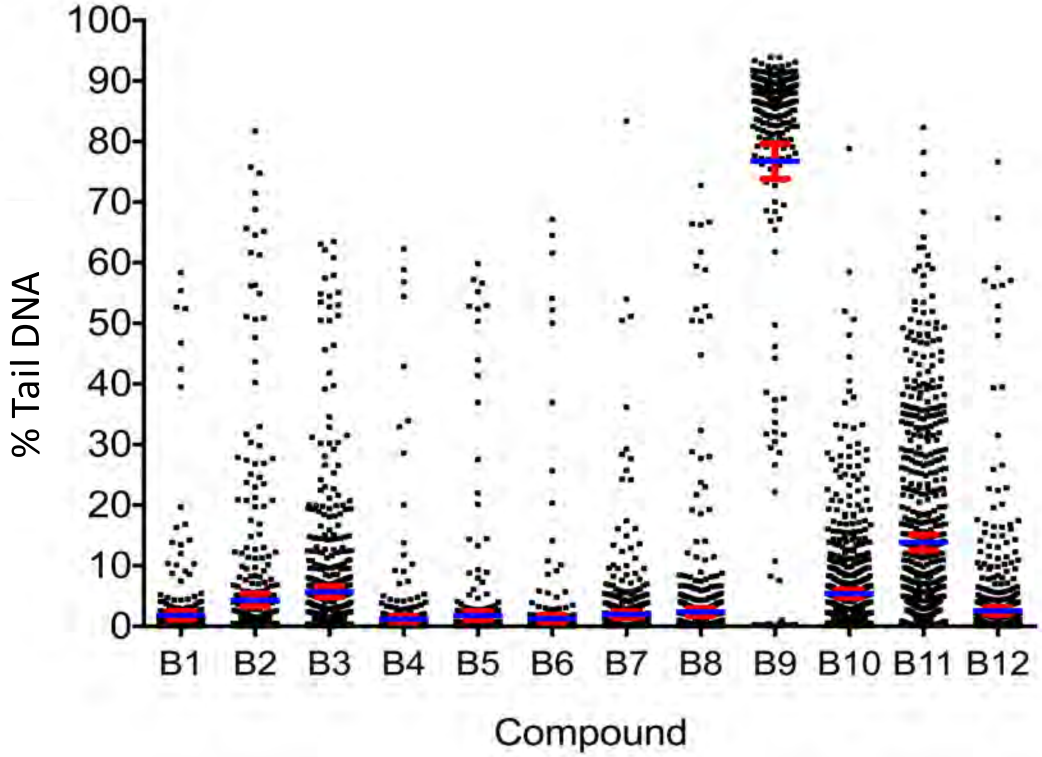

Supplementary Figures – Sykora et al.

Figure S2C

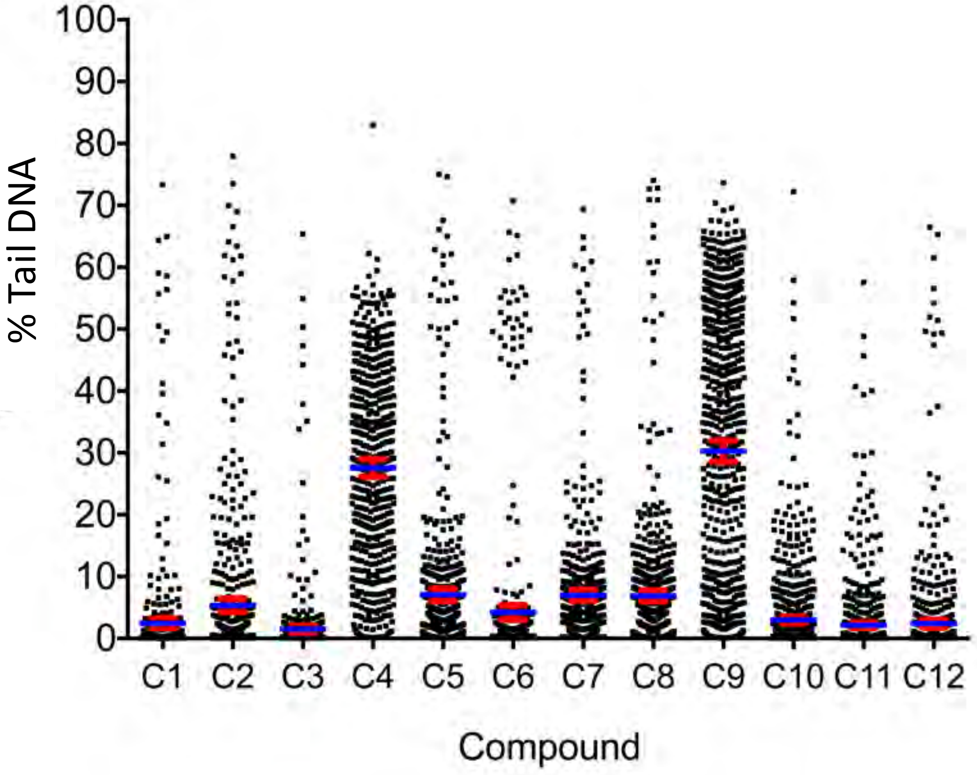

Figure S2D

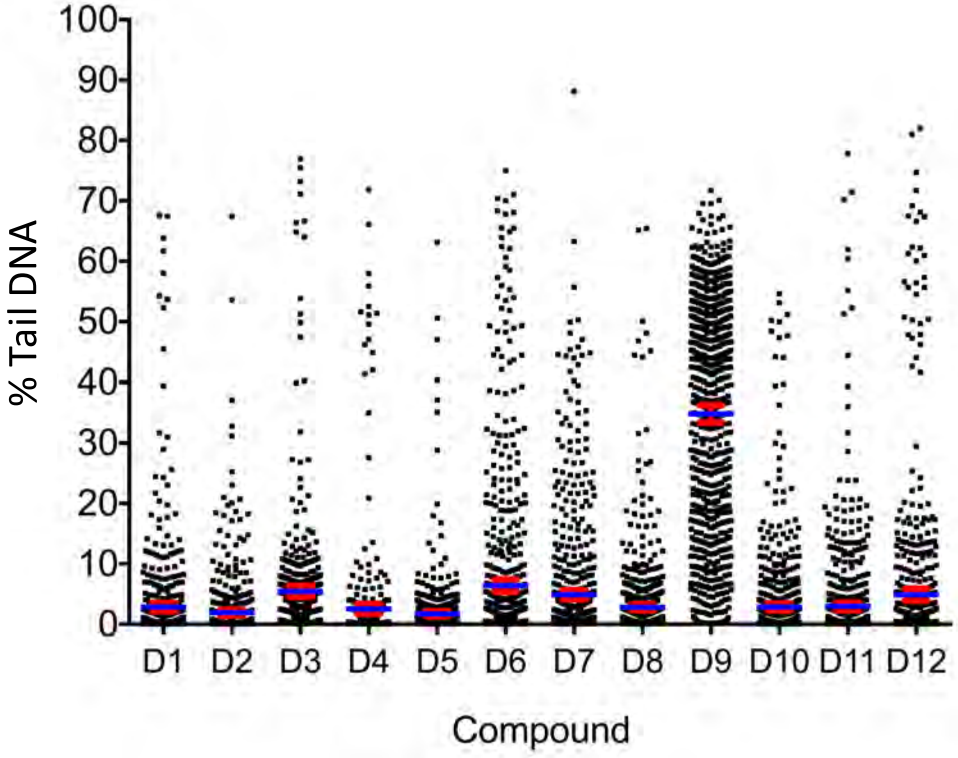

Supplementary Figures – Sykora et al.

Figure S2E

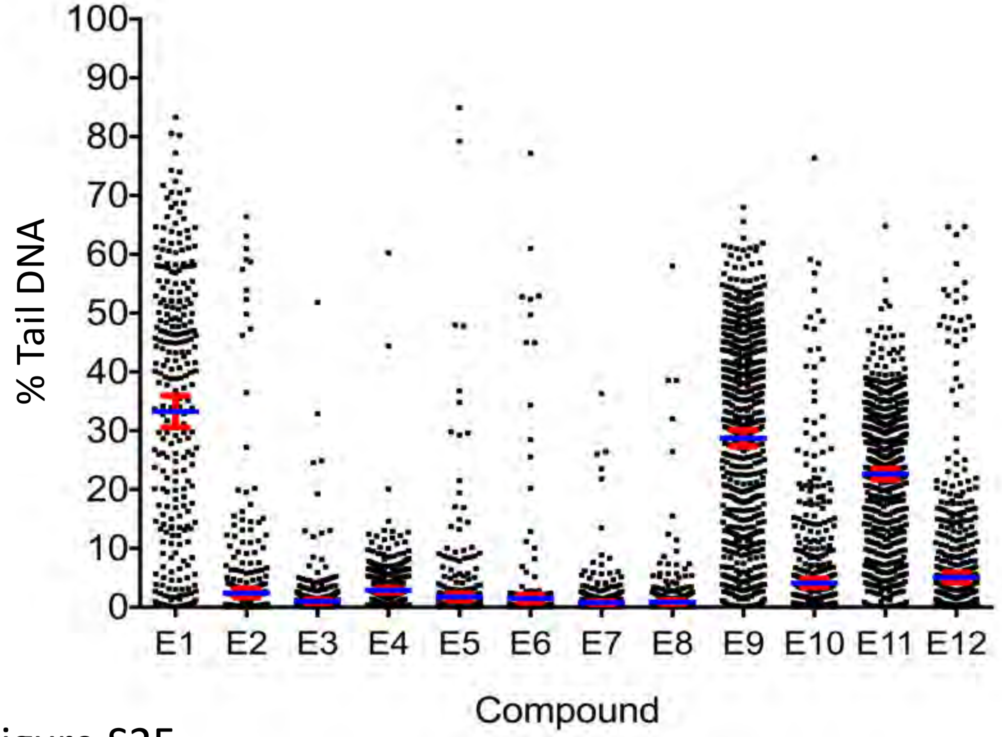

Figure S2F

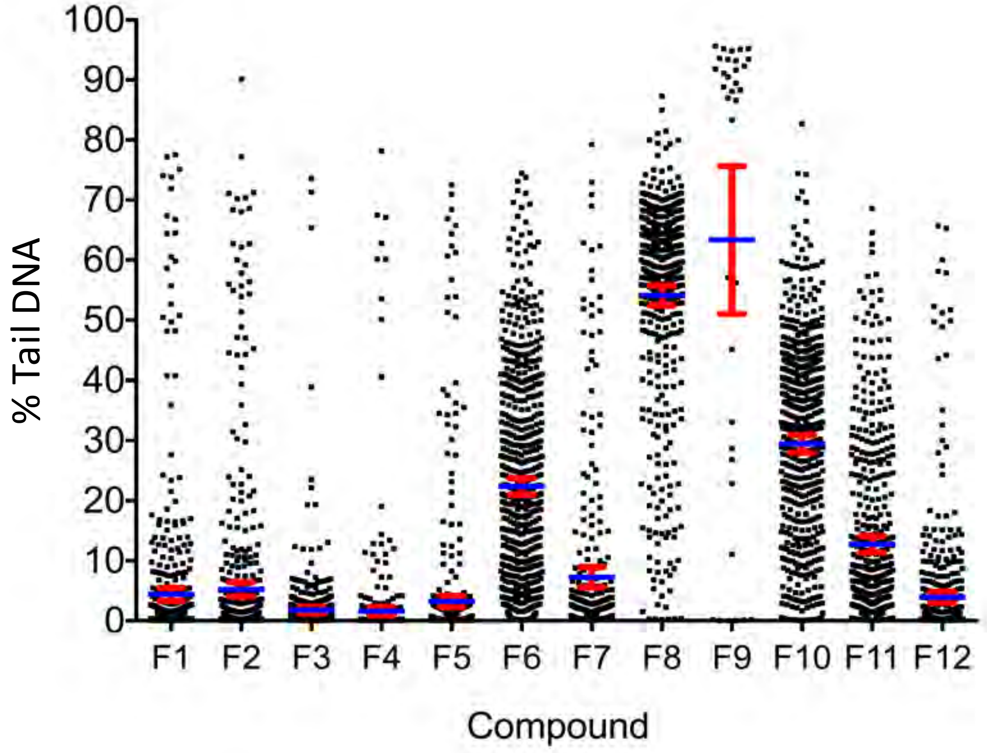

Supplementary Figures – Sykora et al.

Figure S2G

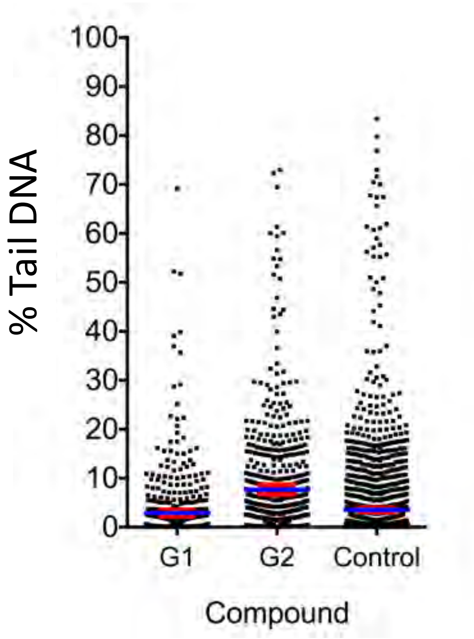

Supplementary Figure S2(A-G) to support Figure 7: Full prescreen data. Jurkat cells were exposed to each compound at a concentration of 100 µM for 1 hr. Alkaline comet was conducted immediately after exposure. Control (NT) cells were vehicle treated. Also see Figure 7. Error bars represent mean ± 95% CI.

Supplementary Figures – Sykora et al.

Figure S3A

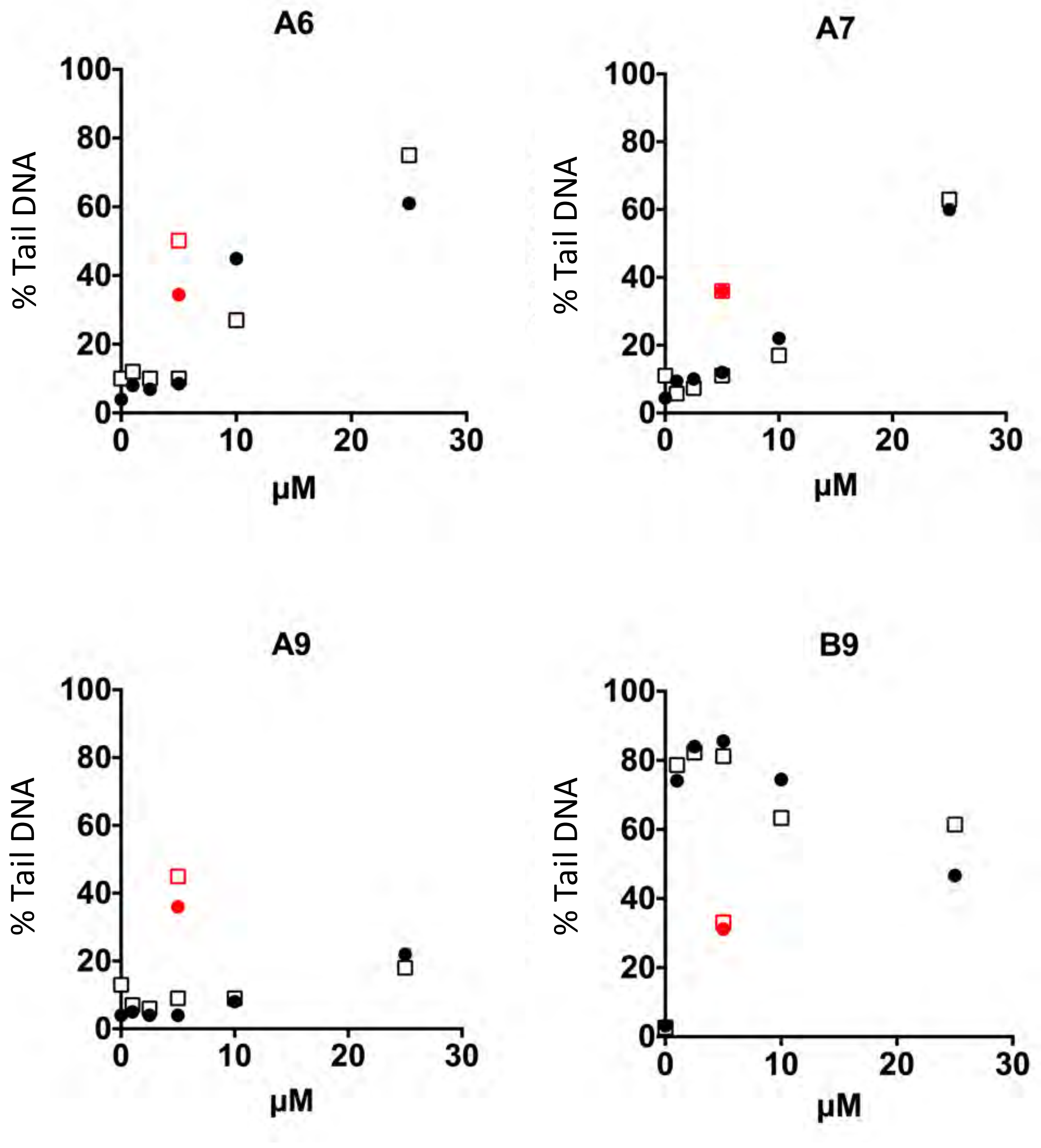

Supplementary Figures – Sykora et al.

Figure S3B

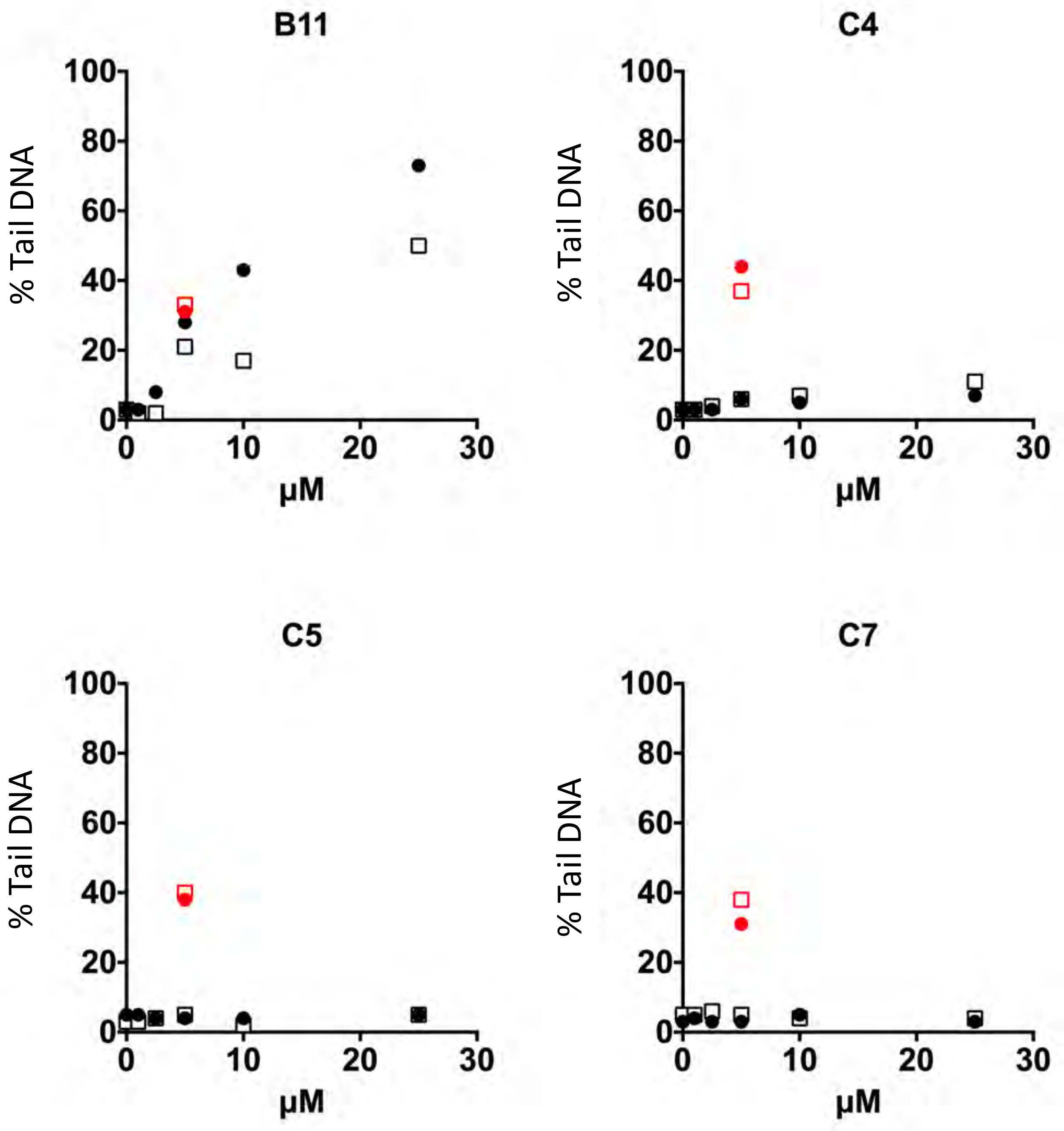

Supplementary Figures – Sykora et al.

Figure S3C

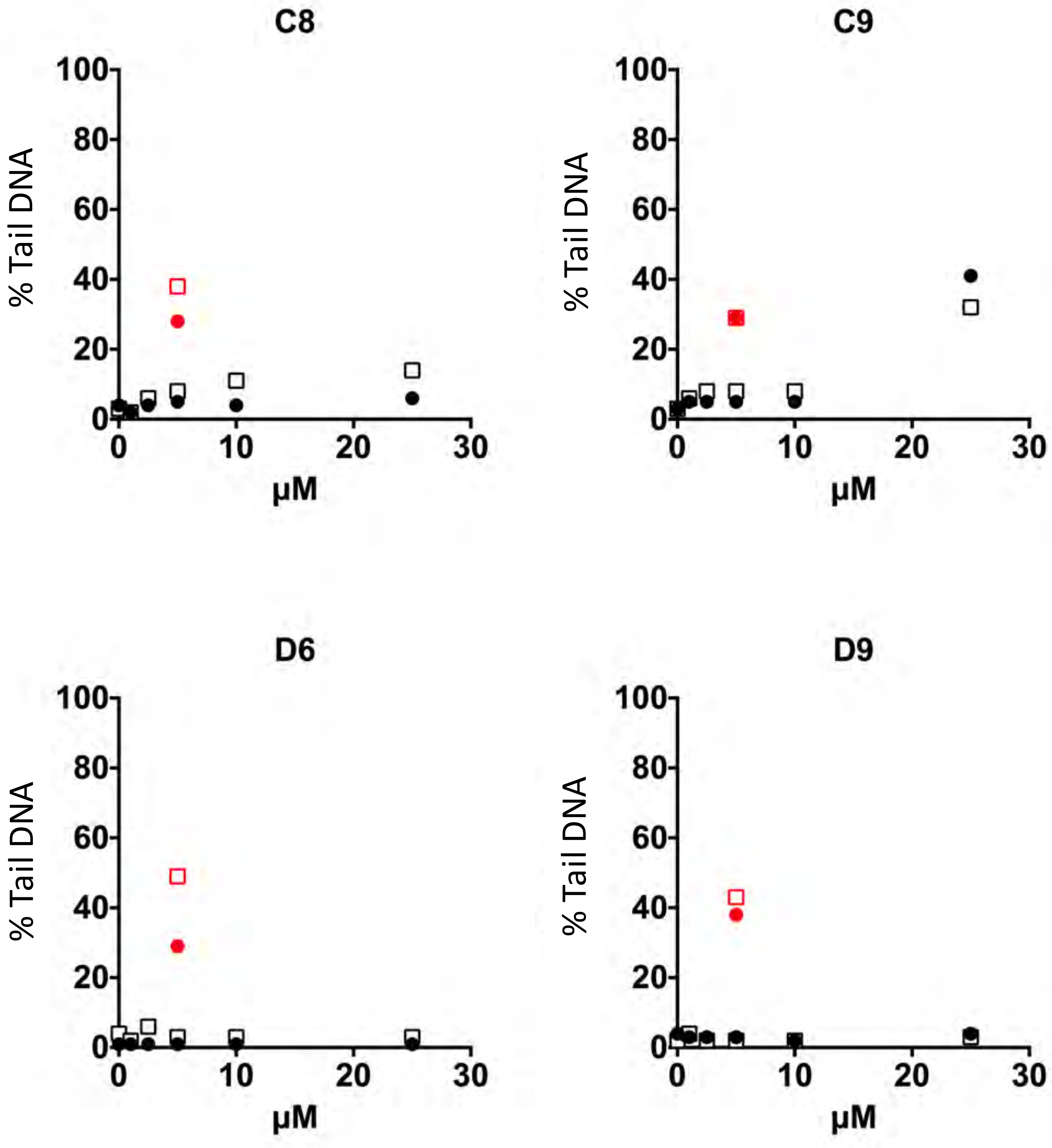

Supplementary Figures – Sykora et al.

Figure S3D

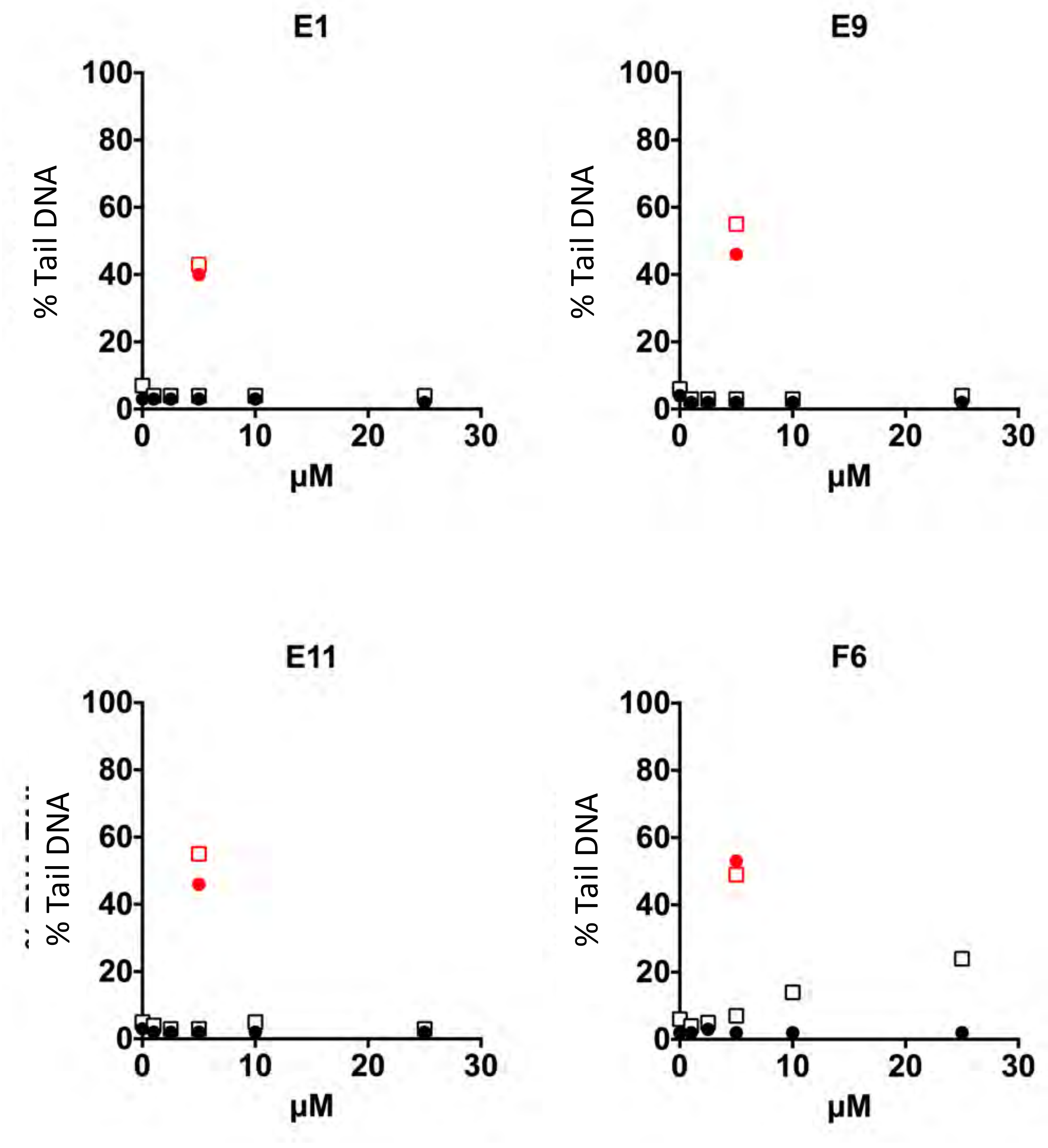

Supplementary Figures – Sykora et al.

Figure S3E

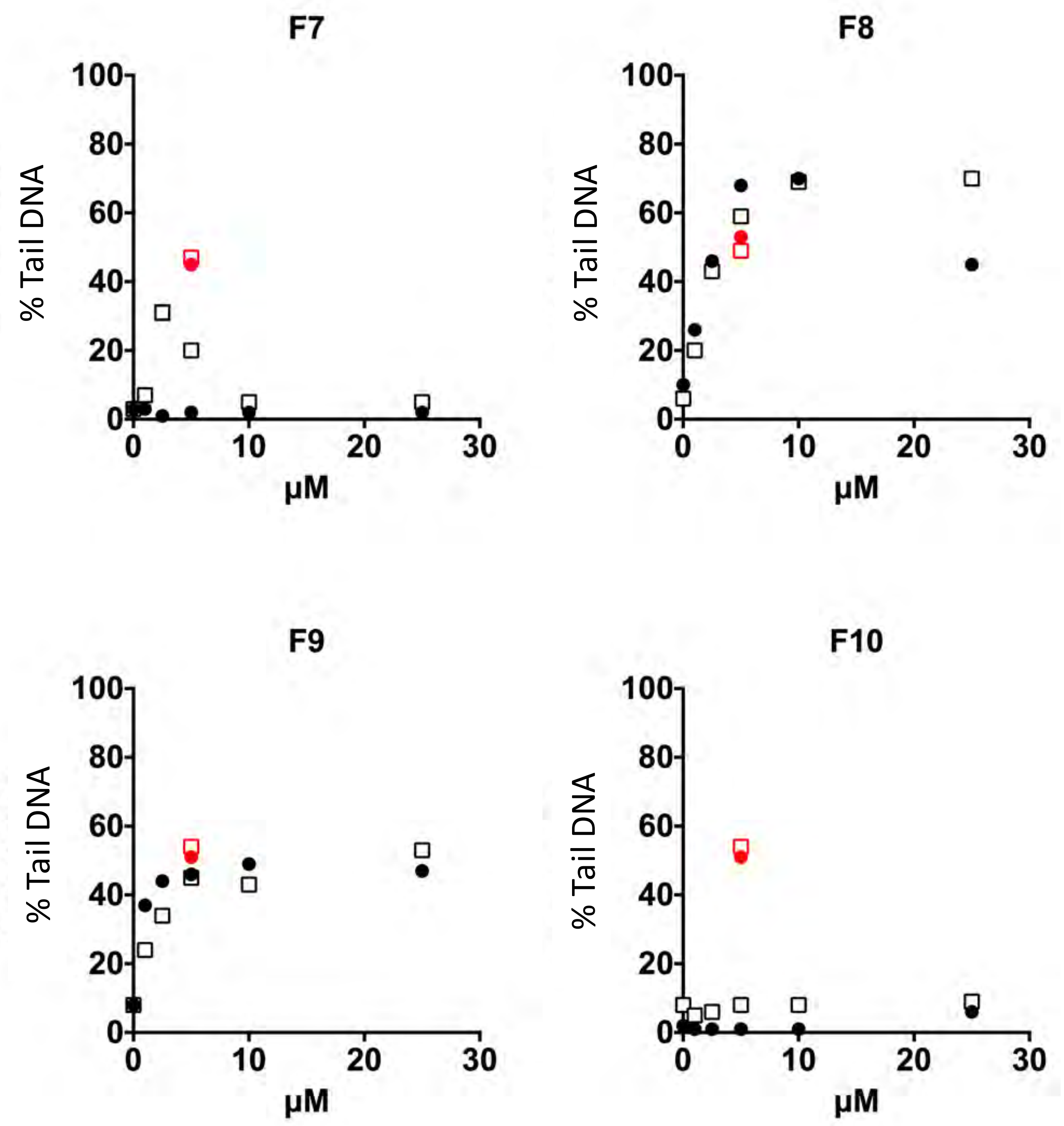

Supplementary Figures – Sykora et al.

Figure S3F

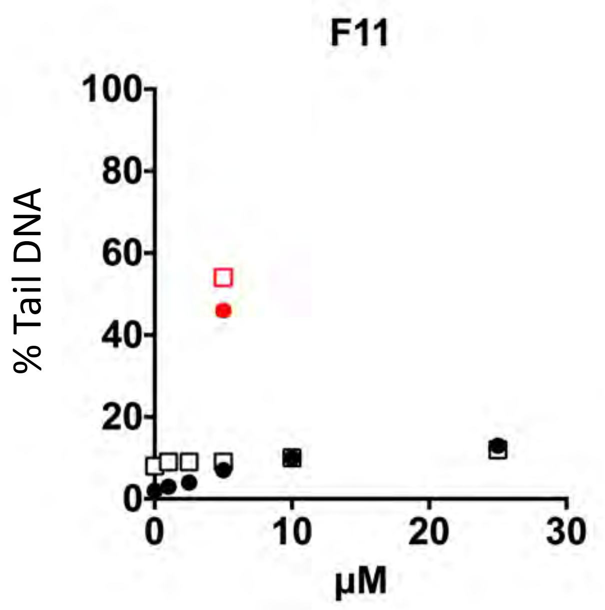

Supplementary Figure S3 (A-F) to support Figure 7: Full screen data from the compounds identified as potentially genotoxic from the prescreen data (Figure 7). Full screen was undertaken using Jurkat (circle) and TK6 (square) cells. Red symbols indicate the positive control (etoposide at 5µM) for each cell line.

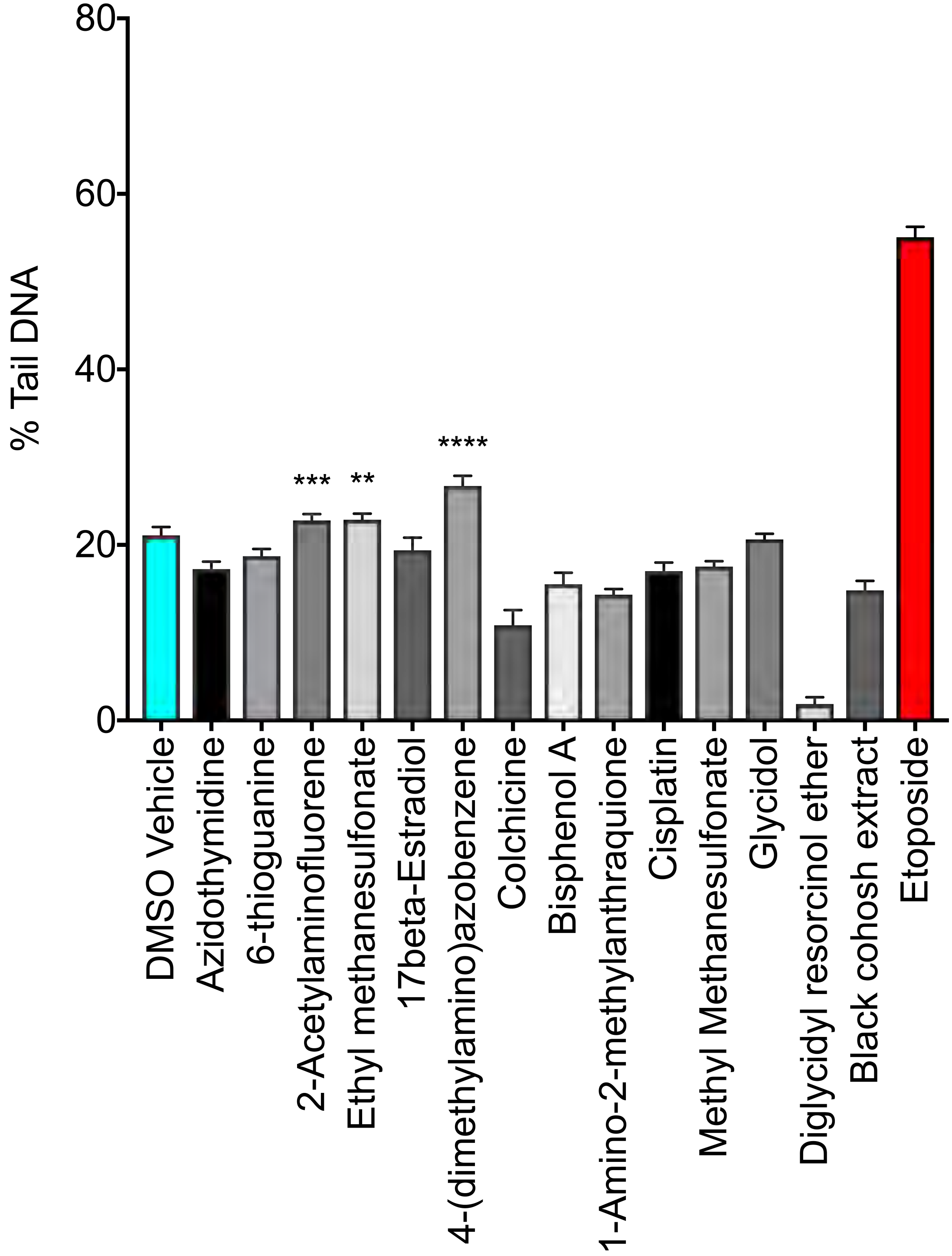

Supplementary Figure S4: TK6 cells were exposed to the non-DNA damaging agents listed in Table 2 for 24 hours. Assay was modified to detect extremely small amounts of DNA damage. Three agents, 4-(dimethylamino)azobenzene, ($p<0.0001$ =****), 2-Acetylaminoflurone ($p<0.001$= ***), Ethyl methanesulfonate ($p<0.01$= **) caused increases in DNA damage. One-way t-test compared to DMSO vehicle control. Etoposide was used as a positive control. n=2 biological replicates, n=4 technical replicates.
